# Novel FAK inhibitors suppress tumor growth and reverse EGFR-TKI resistance in non-small cell lung cancer

**DOI:** 10.20517/cdr.2025.139

**Published:** 2025-11-05

**Authors:** Geng Xu, Camilla Pecoraro, Mahrou Vahabi, Dongmei Deng, Andrea Cavazzoni, Hamid Fiuji, Costanza Anna Maria Lagrasta, Stella M. Cascioferro, Marcello Tiseo, Daniela Carbone, Amir Avan, Paolo A. Zucali, Yehuda G. Assaraf, Godefridus J. Peters, Patrizia Diana, Elisa Giovannetti

**Affiliations:** ^1^Department of Medical Oncology, Cancer Center Amsterdam, Amsterdam UMC, Vrije Universiteit University, Amsterdam 1081 HV, the Netherlands.; ^2^Department of Preventive Dentistry, Academic Centre for Dentistry Amsterdam (ACTA), University of Amsterdam and Vrije Universiteit Amsterdam, Amsterdam 1081 LA, the Netherlands.; ^3^Department of Biological, Chemical, and Pharmaceutical Sciences and Technologies (STEBICEF), University of Palermo, Palermo 90128, Italy.; ^4^Department of Medicine and Surgery, University of Parma, Parma 43126, Italy.; ^5^Metabolic Syndrome Research Center, Mashhad University of Medical Sciences, Mashhad 9177899191, Iran.; ^6^Department of Oncology and Hematology, Medical Oncology Unit, University Hospital of Parma, Parma 43126, Italy.; ^7^Faculty of Health, School of Biomedical Sciences, Queensland University of Technology (QUT), Brisbane 4059, Australia.; ^8^Department of Oncology, IRCCS Humanitas Research Hospital, Rozzano 20089, Italy.; ^9^Department of Biomedical Sciences, Humanitas University, Pieve Emanuele 20072, Italy.; ^10^The Fred Wyszkowski Cancer Research Laboratory, Faculty of Biology, Technion-Israel Institute of Technology, Haifa 3200003, Israel.; ^11^Department of Biochemistry, Medical University of Gdansk, Gdansk 80-211, Poland.; ^12^Cancer Pharmacology Lab, Fondazione Pisana per la Scienza, San Giuliano 56017, Italy.

**Keywords:** Focal adhesion kinase (FAK), EGFR-TKI resistance, non-small cell lung cancer (NSCLC), Afatinib, Osimertinib

## Abstract

**Aim:** The current study aims to investigate the critical role of the focal adhesion kinase (FAK) oncogenic signaling pathway in mediating drug resistance to epidermal growth factor receptor (EGFR)-tyrosine kinase inhibitors (EGFR-TKIs) and evaluate the potential of two novel FAK inhibitors, 10k and 10l, as therapeutic strategies for drug resistant non-small cell lung cancer (NSCLC).

**Methods:** EGFR-TKI resistance in NSCLC cells was developed via stepwise drug selection. Kinases/polymerase chain reaction (PCR) arrays identified key resistance determinants, while reverse transcription quantitative polymerase chain reaction (RT-qPCR), enzyme-linked immunosorbent assay (ELISA), and immunohistochemistry evaluated FAK messenger RNA and phosphorylation levels. Antitumor activities were assessed using sulforhodamine-B, clonogenic, wound-healing, and apoptosis assays, spheroids and xenografts.

**Results:** FAK was identified as a key driver of acquired resistance to EGFR-TKIs. High FAK expression predicted poor prognosis in patients treated with EGFR-TKIs. Kinase and PCR profiling confirmed elevated FAK levels as a resistance mechanism. Compounds 10k and 10l reduced phosphorylated FAK and showed strong anti-proliferative, anti-migratory, and pro-apoptotic effects in both EGFR-TKI-sensitive and -resistant cells. Notably, these compounds were shown to resensitize resistant NSCLC cells to EGFR-TKIs, with 10k inhibiting tumor growth and enhancing Osimertinib efficacy in resistant xenografts.

**Conclusion:** These findings not only uncover a pivotal mechanism of EGFR-TKI drug resistance but also highlight innovative, promising therapeutic options for patients with therapy-refractory disease.

## INTRODUCTION

Lung cancer is one of the most common malignant neoplasms worldwide and the leading cause of cancer-related mortality^[1]^. Non-small cell lung cancer (NSCLC) is the main pathological type of lung cancer, with approximately 30% of cases diagnosed at advanced stages with a dismal 5-year survival rate < 10%^[2]^. The discovery of oncogenic drivers in advanced NSCLC, particularly the epidermal growth factor receptor (EGFR), has paved the way for the development of tyrosine kinase inhibitors (TKIs), which significantly improve progression-free survival in NSCLC patients^[3]^.

Several EGFR-TKIs have been approved for the treatment of NSCLC patients harboring EGFR mutations^[3]^. Unlike the reversible first generation (gefitinib/erlotinib), the second-generation EGFR-TKI Afatinib is an irreversible inhibitor; however, acquired resistance still commonly occurs after initial efficacy^[4]^. In approximately 50% of patients, drug resistance emerges due to the acquisition of the “gatekeeper” mutation T790M, leading to the spatial blockade of first or second-generation TKI binding. Although the third-generation Osimertinib can inhibit T790M, resistance and relapse still occur, with mechanisms including EGFR-dependent (e.g., C797S) and EGFR-independent [e.g., MET proto-oncogene, receptor tyrosine kinase (MET) amplification]^[5,6]^. After resistance, the available options are limited and the eligible population is small^[5,7]^. Therefore, there is an urgent need to develop new therapies to overcome EGFR-TKI resistance.

Focal adhesion kinase (FAK), encoded by the protein tyrosine kinase 2 (*PTK2*) gene, is a cytoplasmic non-receptor tyrosine kinase that plays a crucial role in cancer cell adhesion, survival, proliferation, DNA repair and metastasis through its cross-linked processes with Src, integrin and growth factor receptor signaling pathways^[8,9]^. FAK is often overexpressed and activated in some cancers and elevated levels of FAK are associated with poor prognosis in several cancers^[9,10]^. Notably, EGFR activates FAK through interactions with signaling molecules such as Src kinase. Activation of Src kinase leads to phosphorylation of FAK to promote its binding with other signaling molecules [such as growth factor receptor-bound protein 2 (Grb2), phosphatidylinositol 3-kinase (PI3K), *etc.*], further activating several downstream signaling pathways^[11-13]^. Therefore, targeting FAK can not only inhibit the progression of NSCLC, but may also reverse the acquired resistance of EGFR-TKI^[13,14]^. There is, however, a divergence in findings regarding FAK expression in EGFR-TKI resistant cell lines; some studies^[15]^ have reported high expression, whereas surprisingly, others^[16]^ have found low expression of FAK in EGFR-TKI resistant cell lines. Although FAK expression does not generally correlate with the overall survival (OS) of lung cancer patients^[17-19]^, no research has specifically investigated its association with OS in patients receiving EGFR-TKI treatment. Therefore, the relationship between FAK and EGFR-TKI resistance warrants further exploration.

Several FAK inhibitors have entered clinical trials as monotherapies or in combination with chemotherapy and other targeted therapies; however, progress has been slow, with some trials ending due to suboptimal outcomes^[20,21]^. Although the most promising FAK inhibitor, Defactinib, has been approved for combination therapy with Avutometinib in low-grade serous ovarian cancer, it has not shown clear efficacy in clinical trials for other cancers, including NSCLC^[20,22,23]^. Therefore, exploring new FAK inhibitors is of significant preclinical and clinical interest.

This study integrated data from patients treated with EGFR-TKI and kinase/polymerase chain reaction (PCR) arrays of two drug-resistant cell lines [PC9 Afatinib resistant (PC9AR), H1975 Osimertinib resistant (H1975OR)]. The results showed that in The Cancer Genome Atlas (TCGA)-NSCLC database, FAK was not related to OS, but in our NSCLC patients receiving EGFR-TKI cohort, high FAK expression indicated poor prognosis; resistant cells likewise showed FAK upregulation, and FAK was identified as a resistance “hub” gene. Our newly synthesized FAK inhibitors 10k/10l [10k: 3-(6-Furan-2-yl-imidazo[2,1-b][1, 3, 4]thiadiazol-2-yl)-1H-indole; 10l: 3-(6-Furan-2-yl-imidazo[2,1-b][1, 3, 4]thiadiazol-2-yl)-1-methyl-1H-indole] significantly inhibited proliferation across multiple models^[24]^, showed synergy with EGFR-TKI in resistant cells, and 10k suppressed tumors and restored sensitivity to Osimertinib in a drug-resistant xenograft model.

## METHODS

### Generation of drug-resistant tumor cells

PC9 Afatinib-resistant (AR) and H1975 Osimertinib-resistant (OR) sublines were generated through stepwise drug selection over 6-8 months. Drug concentrations were gradually increased until stable resistance developed. Half-maximal inhibitory concentration (IC_50_) values were periodically assessed, and cells were drug-free for at least 2 weeks before experiments.

Further information on Cell cultures, drugs and reagents, as well as on Cell mutations and copy number analyses is reported in the Supplementary Methods.

### Evaluation of cell growth proliferation using the sulforhodamine B assay and pharmacological interaction

Sulforhodamine B (SRB) assay was performed as previously described^[25]^. Cells (5,000/well in 100 μL medium) were seeded in a 96-well plate and incubated overnight. After 72 h drug treatment, cells were fixed with 50% trichloroacetic acid (25 μL/well, 4 °C, 1 h), washed, and air-dried. SRB dye (0.4%, 50 μL/well) was added to each well and incubated for 30 min, and then removed with 1% acetic acid wash. After air-drying, SRB was dissolved in 10 mM Tris base (150 μL/well, 5 min), and absorbance was measured at 490 nm. The pharmacological interaction between 10k or 10l and EGFR-TKI was assessed using the median-effect analysis method, as described previously^[26]^. Additional details, including those for the colony formation assays, can be found in the Supplementary Methods.

### Studies on multicellular spheroids, migration and apoptosis

Multicellular spheroids were formed by seeding 3,000 cells/well in ultra-low attachment (ULA) plates, followed by treatment and imaging. After 4 days, spheroids were dissociated, and cell proliferation was assessed using a resazurin assay. For migration, we used a wound healing assay. Additional details are provided in the Supplementary Methods.

### Enzyme-linked immunosorbent assay for phosphorylated FAK, Akt and apoptosis

Phosphorylated FAK (p-FAK) levels were measured using an enzyme-linked immunosorbent assay (ELISA) kit (Thermo Fisher)^[27]^. Samples from *in vitro* and *in vivo* studies were incubated with detection antibody, followed by horseradish peroxidase (HRP)-conjugated secondary antibody, and absorbance was measured at 450 nm using a BioTek plate reader. Similarly, phosphorylated Akt levels were measured using a specific ELISA assay (Invitrogen), while apoptosis induction was evaluated by Annexin V and Caspase-3 assays, as described in the Supplementary Methods.

### PCR and kinase activity profiling arrays, bioinformatics, docking and *in vivo* studies

Gene expression was evaluated using reverse transcription quantitative polymerase chain reaction (RT-qPCR), PCR arrays, while kinase activity profiling was conducted with PamChip® arrays. Bioinformatics analyses, molecular docking studies and *in vivo* antitumor activity assessments in mouse models were also performed. Detailed descriptions of these methodologies are provided in the Supplementary Methods.

### Patient tissues and immunohistochemistry

Between 2006 and 2020, NSCLC tissue samples were collected from patients at multiple institutions under approved protocols. All cases were confirmed as NSCLC, and patients had received EGFR-TKI treatment. Immunohistochemistry (IHC) was performed to assess p-FAK expression using anti-p-FAK antibodies, followed by staining evaluation based on intensity and positive staining percentage. Samples were classified as “high” or “low” p-FAK based on the median staining score. Detailed methods are provided in the Supplementary Methods.

### Statistical analysis

Experiments were performed in triplicate and repeated twice. Cell migration percentages were calculated from at least six scratches. Data analysis used Excel, SPSS (v19.0), R Studio, and GraphPad Prism (v9.0). Statistical significance was determined by Student’s *t*-test or one-way Analysis of Variance, with *P* < 0.05 considered significant. Normality was assessed through Shapiro-Wilk testing. Survival analysis was conducted using the Kaplan-Meier method with log-rank testing, and FAK expression correlations were assessed by Spearman’s rank test.

## RESULTS

### Establishment of EGFR-TKI resistant cells

**Figure 1:**
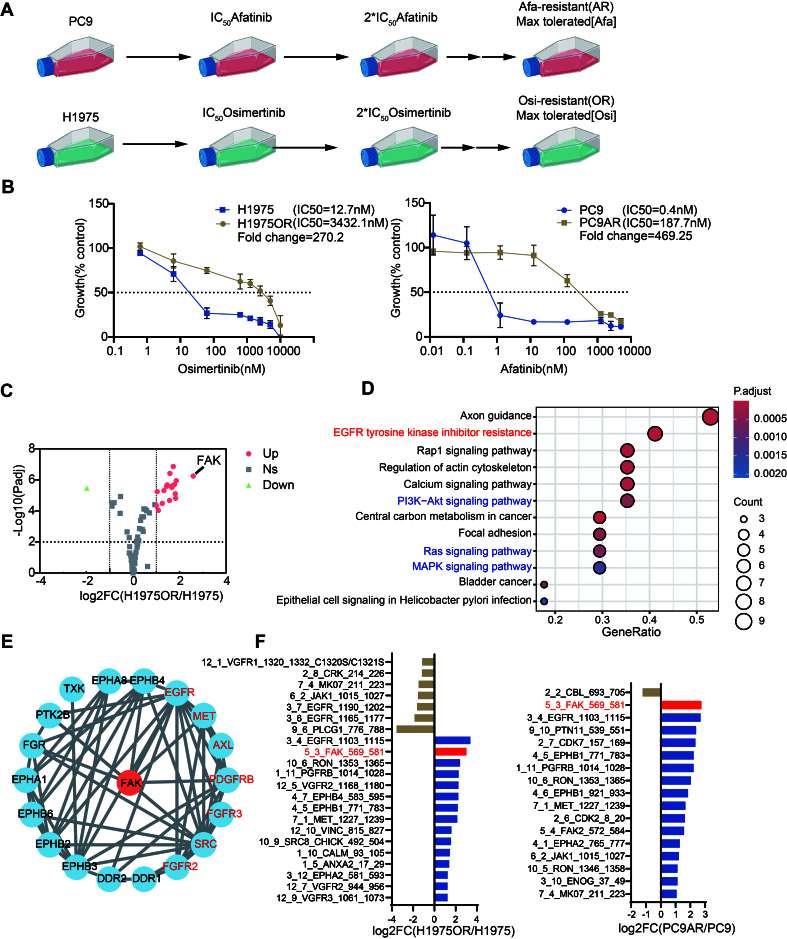
Establishment of NSCLC cell lines resistant to Osimertinib and Afatinib and studies on the key role of FAK in the acquired resistance to EGFR-TKIs in NSCLC. (A) H1975OR and PC9AR cells were established using a gradual stepwise selection protocol as illustrated in the figure [Created in BioRender. Xu, G. (2025)]; (B) H1975/H1975OR and PC9/PC9AR cells were treated with increasing concentrations of indicated drugs (Osimertinib or Afatinib) for 72 h. The growth rate was measured by the SRB assay. (IC_50_ values were calculated by GraphPad Prism and represented as mean ± standard deviation, *n* = 3); (C) H1975 and H1975OR cells were cultured in 6-well plates and harvested for preparation of total RNA and subsequent gene expression analyses. The Volcano plot shows the differential expression of protein kinase genes between H1975 and H1975OR cells. Log2Foldchange > 1 or < -1 and Log10 (*P*adj) > 2 indicate significant differences; (D) KEGG pathway enrichment analysis of significantly DEGs and (E) PPI analysis for DEGs. The genes in EGFR-TKI resistance pathway are depicted in Red; (F) NSCLC cell lines were cultured in 6-well plates and harvested for protein and subsequent PAMchip analyses. The left figure panel shows the difference in phosphorylated protein kinases between H1975 and H1975OR cells. The right panel includes data from PC9 and PC9AR cells. A log2 fold change > 2 was selected as the cutoff value. Kinases with reduced activity are depicted in yellow, while kinases with enhanced activity are in blue; p-FAK is shown in Red. NSCLC: Non-small cell lung cancer; FAK: focal adhesion kinase; EGFR: epidermal growth factor receptor; TKIs: tyrosine kinase inhibitors; H1975OR: H1975 Osimertinib resistant; PC9AR: PC9 Afatinib resistant; SRB: sulforhodamine B; IC_50_: half-maximal inhibitory concentration; KEGG: Kyoto Encyclopedia of Genes and Genomes; DEGs: differentially expressed genes; PPI: protein-protein interaction; p-FAK: phosphorylated focal adhesion kinase; Afa: Afatinib; Osi: Osimertinib; PI3K: phosphatidylinositol 3-kinase; MAPK: mitogen-activated protein kinase. Establishment of NSCLC cell lines resistant to Osimertinib and Afatinib and studies on the key role of FAK in the acquired resistance to EGFR-TKIs in NSCLC. (A) H1975OR and PC9AR cells were established using a gradual stepwise selection protocol as illustrated in the figure [Created in BioRender. Xu, G. (2025)]; (B) H1975/H1975OR and PC9/PC9AR cells were treated with increasing concentrations of indicated drugs (Osimertinib or Afatinib) for 72 h. The growth rate was measured by the SRB assay. (IC_50_ values were calculated by GraphPad Prism and represented as mean ± standard deviation, *n* = 3); (C) H1975 and H1975OR cells were cultured in 6-well plates and harvested for preparation of total RNA and subsequent gene expression analyses. The Volcano plot shows the differential expression of protein kinase genes between H1975 and H1975OR cells. Log2Foldchange > 1 or < -1 and Log10 (*P*adj) > 2 indicate significant differences; (D) KEGG pathway enrichment analysis of significantly DEGs and (E) PPI analysis for DEGs. The genes in EGFR-TKI resistance pathway are depicted in Red; (F) NSCLC cell lines were cultured in 6-well plates and harvested for protein and subsequent PAMchip analyses. The left figure panel shows the difference in phosphorylated protein kinases between H1975 and H1975OR cells. The right panel includes data from PC9 and PC9AR cells. A log2 fold change > 2 was selected as the cutoff value. Kinases with reduced activity are depicted in yellow, while kinases with enhanced activity are in blue; p-FAK is shown in Red. NSCLC: Non-small cell lung cancer; FAK: focal adhesion kinase; EGFR: epidermal growth factor receptor; TKIs: tyrosine kinase inhibitors; H1975OR: H1975 Osimertinib resistant; PC9AR: PC9 Afatinib resistant; SRB: sulforhodamine B; IC_50_: half-maximal inhibitory concentration; KEGG: Kyoto Encyclopedia of Genes and Genomes; DEGs: differentially expressed genes; PPI: protein-protein interaction; p-FAK: phosphorylated focal adhesion kinase; Afa: Afatinib; Osi: Osimertinib; PI3K: phosphatidylinositol 3-kinase; MAPK: mitogen-activated protein kinase.

To investigate the relationships between FAK and EGFR-TKI resistance, we used two commonly used EGFR mutant NSCLC cell lines, PC9 and H1975^[28]^. By exposing these cells to gradually increasing concentrations of Afatinib or Osimertinib for over 6 months, we established EGFR-TKI resistant sublines PC9AR and H1975OR [Figure 1A]. The SRB assay showed that the IC_50_ values of EGFR-TKI resistant cells were hundreds-fold higher than their parental EGFR-TKI sensitive counterpart cells [Figure 1B]. However, we found that PC9AR cells were not resistant to Osimertinib, whereas H1975OR cells were highly resistant to both Osimertinib and Afatinib [Supplementary Figure 1A and B].

Batches of resistant cells used in experiments were maintained in drug-free medium for 2 months and resistance to the maximal tolerated concentration was analyzed by SRB assay at regular intervals. H1975 cells harbor the double *EGFR* exon 21 L858R mutation in cis with the T790M mutation in exon 20, whereas PC9 cells carry an E746-A750 deletion in exon 19 of the *EGFR* gene (c.2235_2249del15). These resistant variants retained their original mutations without acquiring additional *EGFR* mutations, such as the C797S mutation in exon 20 (observed in 10%-26% of cases with second-line Osimertinib resistance cases^[5]^), nor MET gene amplification. These results indicate that these drug resistant model tumor cell lines were genetically stable and constitute a valid model to uncover novel molecular mechanisms of EGFR-TKI resistance.

### *FAK* is a key driver gene of EGFR-TKI resistance in NSCLC

We then analyzed the profiles of oncogenic kinases in EGFR-TKI resistant and sensitive cells. The differentially expressed genes (DEGs) between H1975 and H1975OR cells are depicted in Figure 1C. In particular, we found a total of 16 significantly upregulated genes and 1 significantly downregulated gene in H1975OR cells, with FAK being the most significantly upregulated transcript [Figure 1C]. Figure 1D highlights the various pathways enriched among DEGs through KEGG pathway analysis. Among these pathways, the mitogen-activated protein kinase (MAPK) signaling, PI3K-AKT, and rat sarcoma virus oncogene (RAS) pathways have been identified as significantly associated with EGFR-TKI resistance, consistent with previous studies^[29]^. Additionally, protein-protein interaction (PPI) Network analysis identified FAK as a central or “hub” gene among the DEGs [Figure 1E].

Since the phosphorylated form represents the active state of protein kinases, we used the PamChip array to analyze kinase phosphorylation profiles in EGFR-TKI resistant cells. In particular, the PamChip technology utilizes peptide sequences derived from known kinase substrates, representing various canonical kinase-dependent pathways, as described previously^[30]^. This approach enables the assessment of kinase activity across multiple pathways simultaneously. Our analysis revealed that p-FAK was among the most significantly upregulated kinases in both EGFR-TKI resistant variants [Figure 1F], supporting its potential role in drug resistance mechanisms.

**Figure 2:**
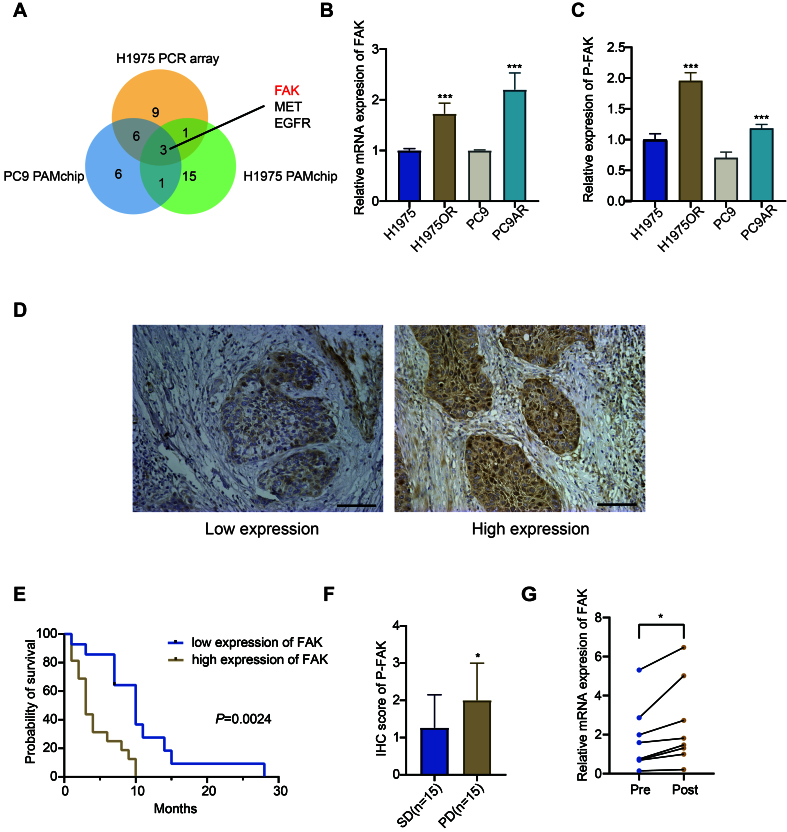
FAK emerges as a biomarker of poor prognosis in NSCLC patients treated with EGFR-TKI. (A) Venn diagram showing genes upregulated in both the PCR array and PAMchip analyses; (B) mRNA expression of FAK was compared between H1975 and H1975OR cells using RT-qPCR. Data were analyzed by DDCt method, as described previously^[51]^; (C) p-FAK levels were measured by ELISA assay in TKI-resistant *vs.* TKI-sensitive cells; (D) Representative IHC images illustrating p-FAK expression in NSCLC patient tissue samples: left panel showing low expression, right panel showing high expression. Scale bars: 100 µm; (E) Survival analysis of NSCLC patients treated with EGFR-TKI, comparing low *vs.* high p-FAK levels; (F) IHC scoring of p-FAK in 30 NSCLC tissue samples. Patients with PD or SD upon EGFR-TKI treatment were compared; (G) Expression of FAK mRNA in 14 NSCLC biopsy samples, before (pre) and after (post) treatment. Data were analyzed by a standard curve method. Statistical significance: *P*-values were set as follows: ^*^P < 0.05, ^***^P < 0.001. The graphs were created with GraphPad Prism. FAK: Focal adhesion kinase; NSCLC: non-small cell lung cancer; EGFR: epidermal growth factor receptor; TKI: tyrosine kinase inhibitor; PCR: polymerase chain reaction; mRNA: messenger RNA; H1975OR: H1975 Osimertinib resistant; RT-qPCR: reverse transcription quantitative polymerase chain reaction; DDCt: Delta Delta Ct; p-FAK: phosphorylated focal adhesion kinase; ELISA: enzyme-linked immunosorbent assay; IHC: immunohistochemistry; PD: progressive disease; SD: stable disease; MET: MET proto-oncogene, receptor tyrosine kinase.

To further understand the functional enrichment of resistance mechanisms, Gene Set Enrichment Analysis (GSEA) was applied. GSEA is a computational method that determines whether an a priori defined set of genes displays statistically significant differences between two biological states - in this case, between EGFR-TKI sensitive and drug resistant cells. This GSEA analysis indicated a significant activation of EGFR-TKI resistance-related pathways in the H1975OR cells [Supplementary Figure 2A]. Specifically, GSEA enrichment identified several genes within the EGFR-TKI resistance axis, as shown in Supplementary Figure 2B. This enrichment of genes underscores their contribution to resistance phenotypes, providing potential therapeutic targets. Furthermore, an intersection analysis of the PamChip phosphorylation data with the gene expression results identified EGFR, MET, and FAK as the sole genes exhibiting upregulated expression at both the transcript and protein phosphorylation levels across the two TKI-resistant tumor cell line models [Figure 2A]. Consistently, the MET gene has been reported to be implicated in EGFR-TKI resistance in multiple studies^[31,32]^.

The close association between genes enriched in the EGFR-TKI resistance pathway and FAK suggests that FAK might orchestrate critical resistance-associated pathways. This supports the hypothesis that FAK plays a pivotal role in mediating resistance mechanisms in EGFR-TKI-treated cells, potentially offering new avenues for therapeutic intervention to overcome resistance.

Next, RT-qPCR analysis confirmed that the *FAK* gene was highly expressed in EGFR-TKI resistant cell lines [Figure 2B]. To validate these findings at the active protein level, an ELISA assay was conducted, confirming the increased levels of p-FAK in the EGFR-TKI resistant cell lines [Figure 2C].

### FAK expression is upregulated in NSCLC patients and is correlated with poor prognosis of NSCLC patients treated with EGFR-TKI

The expression of FAK in NSCLC tissues was initially analyzed in the TCGA database, where we found that FAK messenger RNA (mRNA) levels were significantly higher in LUAD tissue [Supplementary Figure 3A] and LUSC tissue [Supplementary Figure 3B] compared to normal tissue. However, using TCGA-lung adenocarcinoma (LUAD) [Supplementary Figure 3C] and TCGA-lung squamous cell carcinoma (LUSC) [Supplementary Figure 3D] data, survival analysis indicated that FAK mRNA levels were not associated with OS in NSCLC patients. Notably, in this dataset, patients were not selected for the presence of EGFR mutations or specific treatment with EGFR-TKIs, and the overall conclusion from this analysis is that FAK mRNA levels are not a prognostic marker in NSCLC.

To further investigate the specific clinical impact of p-FAK in patients treated with EGFR-TKIs, we conducted IHC analysis in a cohort of NSCLC patients who received EGFR-TKI therapy. Table 1 depicts the characteristics of this cohort, and Figure 2D shows representative images of low and high p-FAK expression in two representative tissues. In this cohort, factors such as gender, age, smoking history, and pathological type were not found to be associated with OS [Supplementary Table 1]. However, survival analysis revealed a negative correlation between p-FAK levels and OS in patients treated with EGFR-TKI (*P* = 0.0024; Figure 2E). In addition to p-FAK, the performance status (PS) and the stage of NSCLC were identified as significant risk factors for OS [Supplementary Table 1]. However, we did not observe a correlation between p-FAK levels and these, or other clinical-pathological features of lung cancer, except for the observation that older patients exhibited lower p-FAK expression, as reported in Table 1. This finding was unexpected and has not been previously reported; however, age was not correlated with FAK expression nor response to EGFR-TKIs in previous studies^[33,34]^. Remarkably, p-FAK was identified not only as a marker for OS, but also as a predictor of treatment response. Among the 30 patients treated with EGFR-TKIs, p-FAK levels were significantly higher in the progressive disease (PD) group compared to the stable disease (SD) group [Figure 2F].

**Table 1:** Association between p-FAK expression and clinicopathological features of lung cancer

**Characteristic**	**Patients**	**p-FAK expression**		***P*-value**
	***n* (%)**	**Low expression**	**High expression**	
Gender				0.355
Male	21 (70)	11	10	
Female	9 (30)	3	6	
Age (years)				0.031^*^
≤ 65	22 (73)	9	13	
> 65	8 (27)	5	3	
Stage				0.064
I/II	8 (27)	6	2	
III/IV	22 (73)	8	14	
PS				0.094
0/1	27 (90)	14	13	
2	3 (10)	0	3	
Smoking				0.529
Yes	25 (83)	11	14	
No	5 (17)	3	2	
Pathological type				0.529
SPINO	7 (23)	4	3	
ADC	21 (70)	9	12	
NAS	2 (7)	1	1	

Statistical significance: *P*-values was set as follows: ^*^P < 0.05. p-FAK: Phosphorylated focal adhesion kinase; PS: performance status; SPINO: squamous cell spinocarcinoma; ADC: adenocarcinoma; NAS: not otherwise specified.

Lastly, we analyzed the modulation of FAK mRNA levels in 7 patients before and after EGFR-TKI treatment. Despite the limited sample size, pre-treatment FAK mRNA levels in these patients correlated with p-FAK levels [Supplementary Figure 4]. Notably, in all these patients who experienced disease progression post-treatment, FAK transcript levels were significantly elevated [Figure 2G]. These findings suggest that increased FAK levels may serve as a potential monitoring biomarker for acquired resistance to EGFR-TKIs in serial biopsies from NSCLC patients.

**Figure 3:**
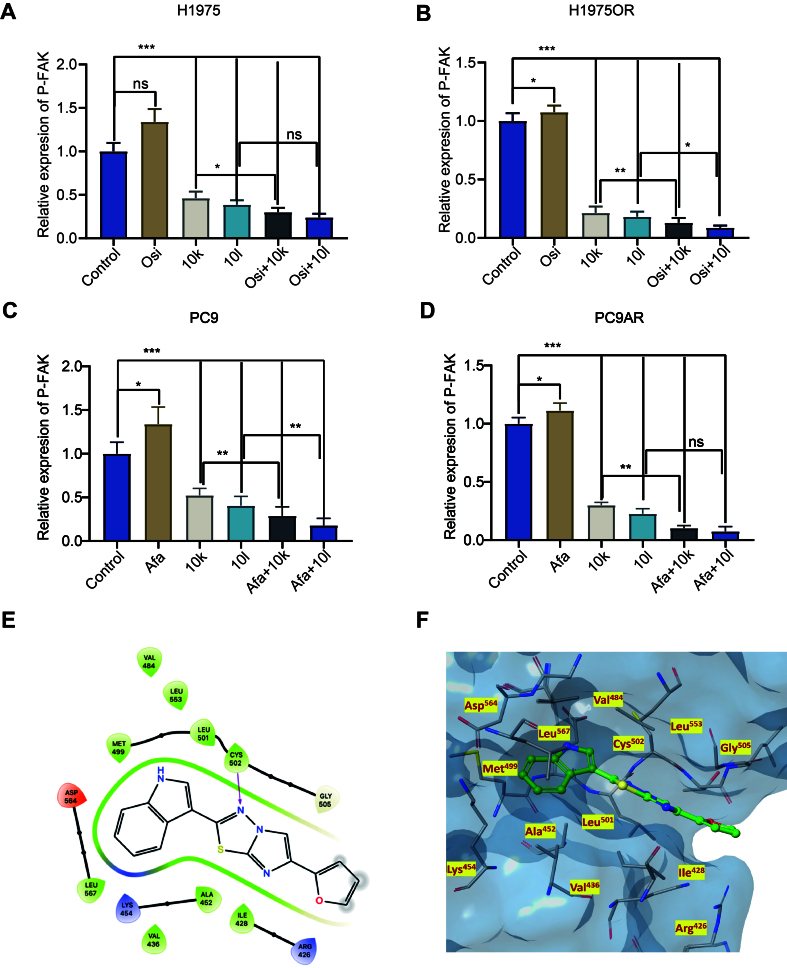
Structure and kinase inhibitory activities of the new FAK inhibitors. (A-D) NSCLC cells (PC9, PC9AR, H1975, and H1975OR) were treated with Afatinib, Osimertinib, 10k, 10l, or their combinations for 24 h, followed by protein extraction. The levels of p-FAK were measured using an ELISA assay. NSCLC Cells were treated with each compound at concentrations corresponding to their respective IC_50_ values for the indicated cell lines; (E) 2D ligand interaction diagram for compound 10k; (F) 3D complex of the FAK catalytic domain (PDB code: 6YOJ) with compound 10k; nitrogen, oxygen, and sulfur atoms are shown in blue, red, and yellow, respectively. Statistical significance: *P*-values were set as follows: ^*^P < 0.05, ^**^P < 0.01, ^***^P < 0.001, “ns” indicates a non-significant difference. The graphs were created with GraphPad Prism. FAK: Focal adhesion kinase; NSCLC: non-small cell lung cancer; PC9AR: PC9 Afatinib resistant; H1975OR: H1975 Osimertinib resistant; P-FAK: phosphorylated focal adhesion kinase; ELISA: enzyme-linked immunosorbent assay; IC_50_: half-maximal inhibitory concentration; 2D: two-dimensional; 3D: three-dimensional; PDB: Protein Data Bank; Afa: Afatinib; Osi: Osimertinib.

Notably, p-FAK expression levels were found to be consistently elevated following treatment with EGFR-TKI also in our NSCLC models, as shown in Figure 3A-D. Drug resistance in cancer cells is often linked to dynamic changes in gene expression that occur as an adaptive response to therapeutic agents. In drug-sensitive cells, initial exposure to a drug may induce a transient upregulation of certain genes as part of a stress response. However, when gene upregulation persists and becomes stabilized in drug-resistant variants, it suggests that the gene may play a protective role, facilitating cellular adaptation and survival in the presence of the drug^[35,36]^. Therefore, we hypothesize that the persistent upregulation and activation of FAK are the result of signaling cascades promoting cellular survival in drug-resistant cells, as described in previous studies in platinum and taxane-resistant tumors^[37,38]^. This prompted the following studies with new FAK inhibitors.

### The structure and kinase inhibitory activities of the new FAK inhibitors 10k and 10l

In a previous study, we synthesized imidazo[2,1-b][1,3,4]thiadiazole derivatives as FAK inhibitors^[24]^, and their chemical structures are shown in Supplementary Figure 5A. We initially tested these compounds in pancreatic cancer cell lines, where derivatives 10k and 10l exhibited significant cytotoxic activity. In the current study, we tested the antitumor activities of 10k and 10l in NSCLC cells. Additionally, we also performed additional molecular docking studies to investigate the binding modes of 10k and 10l to the FAK protein. This analysis revealed that both derivatives can bind to the catalytic domain of FAK, interacting with key amino acids at the binding site. In particular, derivative 10k, with the imidazo[2,1-b][1,3,4]thiadiazole core, interacts with the hinge region by forming hydrogen bonds with the C=O group of the Cys502 residue. Additionally, the gatekeeper residue (Met499) and a solvent region (Arg426, Ile428, Ala452, and Leu567) participate in electrostatic interactions with 10k [Figure 3E and F]. Interestingly, Cys502 was not favored for binding by either of the two inhibitors. However, as shown in Supplementary Figure 5B and C, compound 10l forms a hydrogen bond with Glu506 through the oxygen atom of its furan ring. We then evaluated the inhibitory effects of 10k and 10l on FAK using ELISA assays. Treatment of NSCLC cell lines with 10k and 10l led to a significant reduction in p-FAK levels [Figure 3A-D]. While Osimertinib and Afatinib increased p-FAK expression, the combination of 10k or 10l with these EGFR-TKIs resulted in a more pronounced reduction in p-FAK levels compared to monotherapy treatments [Figure 3A-D]. In conclusion, 10k and 10l markedly reduced p-FAK levels and these findings prompted further investigation to explore whether this inhibition might affect various signaling pathways downstream of FAK, particularly those involved in cell survival and migration.

### The new FAK inhibitors 10k and 10l inhibit cell proliferation and migration in both EGFR-TKI sensitive and resistant tumor cells

The compounds 10k and 10l inhibited cell proliferation in a dose-dependent manner, with IC_50_ values ranging from 2 to 9 μM across PC9, PC9AR, H1975, and H1975OR cell lines [Supplementary Table 2]. The IC_50_ values of both compounds were comparable between EGFR-TKI-sensitive and drug-resistant cell lines [Supplementary Table 2], suggesting that there is no cross-resistance between EGFR-TKI and FAK inhibitors. We also evaluated the anti-proliferative activity of Defactinib, and found that, although 10k exhibited a higher IC_50_ than Defactinib, 10l showed IC_50_ values comparable to Defactinib across all tested NSCLC cell models [Supplementary Table 2]. Additionally, the anticancer activity of 10k and 10l was further assessed in colony formation [Supplementary Figure 6A and B] as well as cell in spheroid assays [Supplementary Figure 6C and D]. Both compounds significantly blocked colony formation and spheroid growth in all EGFR-TKI-sensitive and drug-resistant cells. These results indicate that 10k and 10l effectively inhibit cell proliferation in both 2D and 3D NSCLC cell models.

The FAK signaling pathway plays a crucial role in cell migration across various tumor types, including NSCLC^[9]^. To evaluate the effects of 10k and 10l on cell migration, we conducted a wound healing assay. As shown in Supplementary Figure 6E and F, both 10k and 10l significantly inhibited the migration of PC9, PC9AR, H1975 and H1975OR cells. Additionally, treatment with them led to downregulation of the mRNA levels of a key regulator of cell migration, matrix metalloproteinase-9 (MMP9, Supplementary Figure 7).

### The new FAK inhibitors 10k or 10l increase apoptosis and necrosis of EGFR-TKI sensitive and resistant cells

After treatment with 10k or 10l, we observed significant changes in cell morphology, including cell shrinkage, increased cytoplasmic density, and fragmentation, which are characteristic features of cell death induction [Supplementary Figure 8A]. To determine whether the inhibition of cell proliferation by 10k or 10l was due to apoptosis or necrosis, we evaluated the levels of Annexin V, PI, and BCL2 in NSCLC cell lines. Annexin V is a marker of apoptosis, PI indicates necrosis, whereas BCL2 is a well-established anti-apoptotic protein. Following treatment with 10k or 10l, NSCLC cells were double-stained with Annexin V/PI. Representative images of Annexin V staining levels after drug treatment are shown in Supplementary Figure 8B. We found that both 10k and 10l increased Annexin V and PI levels in all tested cell lines [Supplementary Figure 8C and D]. Additionally, BCL2 mRNA levels were significantly reduced after treatment with 10k or 10l [Supplementary Figure 8E]. We also observed that BCL2 mRNA levels were higher in EGFR-TKI resistant cells compared to EGFR-TKI sensitive parental cells [Supplementary Figure 9]. These findings show that 10k and 10l promote both apoptosis and necrosis in EGFR-TKI sensitive and drug-resistant NSCLC cells.

### The new FAK inhibitors 10k or 10l restore sensitivity of EGFR-TKI resistant cells to Afatinib and Osimertinib

**Figure 4:**
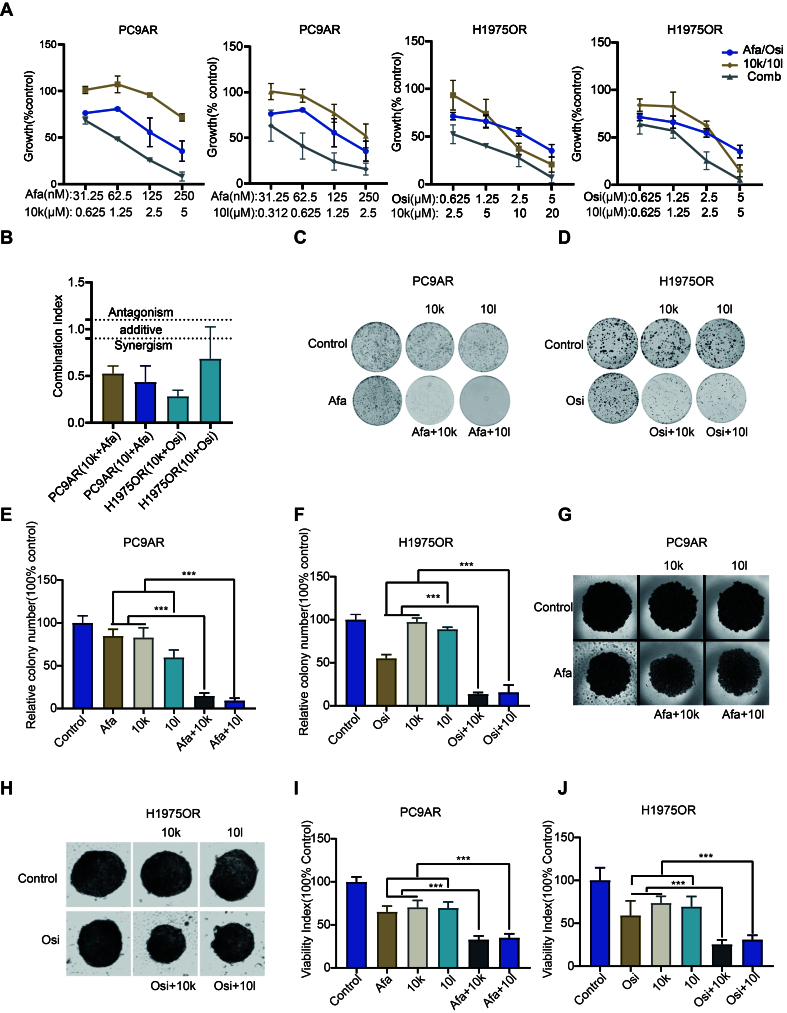
The new FAK inhibitors 10k and 10l enhance EGFR-TKI sensitivity in drug resistant cells. (A) PC9AR and H1975OR cells were treated with the indicated concentrations of the tested drugs, either alone or in combination, and growth rates were measured using the SRB assay after 72 h. The compounds 10k and 10l treated the same cell line in one experiment; (B) CIs at 50 %, 75%, and 90% values of fraction affected revealed a synergistic interaction between the drugs; (C and D) PC9AR and H1975OR cells were plated in 6-well plates (1 × 10^3^ cells/well) and treated for 10 days with indicated compounds. Representative images of colony formation are shown; (E and F) Quantitative analysis of colony formation results. PC9AR cells: treated with Afatinib, 10k, 10l at 0.125 × IC_50_ (PC9AR cells) or combination, H1975OR cells: treated with Osimertinib, 10k, 10l at 0.125 × IC_50_ (H1975OR cells) or combination; (G and H) Spheroids generated from PC9AR and H1975OR cells were treated for 4 days with indicated compounds. PC9AR cells: treated with Afatinib at 0.125 × IC_50_ (PC9AR cells) and 10k or 10l at 1.5 × IC_50_ (PC9AR cells) or combination, H1975OR cells: treated with Osimertinib at 0.125 × IC_50_ (H1975OR cells) and 10k, 10l at 1 × IC_50_ (H1975OR cells) or combination. Representative images of spheroids are shown; (I and J) Viability of spheroids was assessed using the resazurin assay. Statistical significance was set as follows: ^***^P < 0.001. The graphs were created with GraphPad Prism. FAK: Focal adhesion kinase; EGFR: epidermal growth factor receptor; TKI: tyrosine kinase inhibitor; PC9AR: PC9 Afatinib resistant; H1975OR: H1975 Osimertinib resistant; SRB: sulforhodamine B; CIs: combination indices; IC_50_: half-maximal inhibitory concentration; Afa: Afatinib; Osi: Osimertinib.

Our findings indicate that highly activated FAK may contribute to EGFR-TKI resistance. Hence, we examined whether blocking FAK with 10k or 10l can restore the sensitivity of EGFR-TKI resistant cells to Afatinib and Osimertinib. As shown in Figure 4A, the combination of Afatinib and Osimertinib with 10k or 10l was more efficacious in blocking the growth of PC9AR or H1975OR cells at different concentrations, compared with single drug treatments. All combination indices (CIs) of different combinations were very low and significantly below 1, hence indicating a synergistic interaction [Figure 4B].

Moreover, we evaluated the anticancer activity of the drug combinations using colony formation [Figure 4C-F] and the cell spheroid assay [Figure 4G-J]. All drug combinations more effectively blocked colony formation and the growth of cell spheroids compared to single drugs. In particular, the combination of Afatinib and Osimertinib with 10k or 10l was synergistic also in three-dimensional tumor spheroid models. Furthermore, we detected significantly higher levels of Annexin V and PI in EGFR-TKI resistant cells treated with different drug combinations compared to single drug treatments [Supplementary Figure 10A-D]. In summary, these findings demonstrate that the combination of 10k or 10l with EGFR-TKIs markedly enhances the inhibition of cell proliferation and increases the apoptosis and necrosis in EGFR-TKI resistant NSCLC cell models.

### The new FAK inhibitor 10k inhibited the growth of NSCLC cell xenograft models, modulated FAK and downstream pathways favoring apoptosis induction, and enhanced the sensitivity to Osimertinib *in vivo*

**Figure 5:**
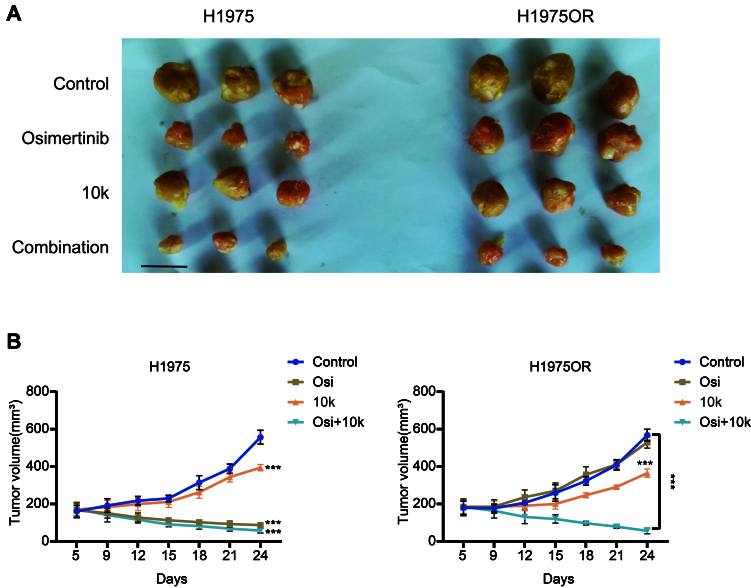
The novel FAK inhibitor 10k enhances the activity of Osimertinib *in vivo*. (A) Tumor-bearing mice with H1975 (left panel) or H1975OR (right panel) xenografts were treated with 10k (20 mg/kg), Osimertinib (3 mg/kg), or a combination of both drugs at the same doses for 2 weeks. After one additional week, tumors were excised from the mice and photographed. Scale bars: 1 cm; (B) Tumor volumes were measured at regular intervals during the treatment. Statistical significance: *P*-values was set as follows: ^***^P < 0.001. The graphs were created with GraphPad Prism. FAK: Focal adhesion kinase; H1975OR: H1975 Osimertinib resistant; Afa: Afatinib; Osi: Osimertinib.

Since 10k displayed a superior CI with Osimertinib compared to 10l, and as Osimertinib is the most commonly administered (third-generation) EGFR-TKI, we selected 10k and Osimertinib for further *in vivo* studies. These studies demonstrated that 10k effectively inhibited tumor cell proliferation and growth in both H1975 and H1975OR xenograft models, as also assessed by reduction of Ki67 staining in H1975OR specimens [Supplementary Figure 11A], while Osimertinib was only able to inhibit the growth of H1975 tumors [Figure 5A and B]. In addition, the data presented in Supplementary Figure 11 show that 10k treatment suppressed the expression of both p-FAK and its downstream effector p-Akt in H1975OR tumors [Supplementary Figure 11B and C]. Consistently, caspase-3 activity assays indicated that 10k increased apoptosis in these tumor tissues [Supplementary Figure 11D]. Defactinib, a well-studied FAK inhibitor, was also tested in the same mouse xenograft model; we found that 10k exhibited similar inhibitory effects to Defactinib [Supplementary Figure 12A] with a Defactinib concentration used in our previous study^[39]^. As shown in Figure 5B, the combination of Osimertinib and 10k demonstrated a stronger inhibitory effect on H1975OR xenograft growth compared to either drug alone. Notably, data reported in Supplementary Figure 12B confirmed that 10k, Osimertinib, Defactinib, and the combination of 10k with Osimertinib did not exert any deleterious effect on the weight of mice at the indicated doses. These findings suggest that 10k effectively suppresses tumor growth *in vivo* and can markedly enhance the sensitivity of Osimertinib in TKI-resistant xenograft models, without causing untoward toxicity.

## DISCUSSION

NSCLC patients can develop resistance to EGFR-TKIs through EGFR-independent mechanisms, such as activation of alternative bypass pathways, aberrant downstream signaling, or histologic transformation^[5,31]^. In the current study, FAK emerged as the most prominently activated protein kinase in two distinct models of EGFR-TKI-resistant NSCLC cells. Furthermore, we observed that both Afatinib and Osimertinib increased p-FAK levels in both EGFR-TKI-resistant and sensitive cells - a novel phenomenon which was not previously reported^[40,41]^. Notably, while some studies have documented increased p-FAK levels in OR H1975 or HCC827 cells^[15]^, others have reported decreased p-FAK expression in gefitinib-resistant HCC827 cells^[16]^. However, these discrepancies align with findings indicating that drug resistance is a multifactorial and complex phenomenon, shaped by the acquisition of distinct molecular mechanisms in different tumor cell lines^[42,43]^. The mechanism by which FAK activation leads to EGFR-TKI resistance is rather complex. For example, evidence exists that abnormally activated osteopontin (OPN)/integrin αVβ3/FAK signaling is responsible for EGFR-TKI resistance^[16]^. Remarkably, Ibrutinib can reverse Osimertinib resistance through inhibition of Laminin α5/FAK signaling^[40]^.

Our analysis of gene and protein kinase profiles using PCR arrays and PamChip revealed that FAK was a central hub gene among the DEGs in drug-resistant cells, and it displayed a strong correlation with genes enriched in the EGFR-TKI resistance pathway. Additionally, the MAPK, PI3K-AKT, and RAS signaling pathways were found to be activated in EGFR-TKI-resistant cells^[29]^. Previous studies have demonstrated that these pathways are closely associated with FAK, further supporting its key role in drug resistance mechanisms^[9,21,29]^.

Several studies have shown that FAK was highly expressed in various cancer types. In addition, FAK upregulation and activation were associated with poor prognosis and aggressive disease progression^[9,10]^. Herein, we confirmed that FAK is significantly expressed in different types of NSCLC tissues (LUAD and LUSC) based on the TCGA dataset. Interestingly, while FAK expression was not correlated with poor prognosis for lung cancer patients in the TCGA dataset, our patient cohort revealed that FAK serves as a marker for prognosis and response to EGFR-TKIs. This can be explained by the fact that the lung cancer cases in the TCGA dataset include a heterogeneous mixture of mutations, with EGFR mutations likely comprising only a small subset and only a small subset of TCGA cases were treated with EGFR-TKIs. Conversely, our current study in NSCLC patients with EGFR mutations undergoing EGFR-TKI therapy, showed significantly higher FAK expression in patients with shorter OS and PD, compared to those with longer OS and SD. Thus, FAK emerges as a prognostic marker for poor outcomes in patients treated with EGFR-TKIs. Furthermore, our findings from repeated biopsies indicate that EGFR-TKI treatment leads to increased FAK expression levels. This elevated FAK expression can contribute to resistance to EGFR-TKIs, ultimately driving disease progression.

These consistent findings of FAK upregulation in EGFR-TKI-resistant cells and tumor tissues not only underscore its role in mediating chemoresistance but also position it as a promising therapeutic target. Targeting FAK or its downstream signaling pathways could potentially reverse resistance and enhance the efficacy of EGFR-TKI treatment. In previous studies, we screened a series of new FAK inhibitors with imidazo[1,2-b]pyridine pharmacophore structures, thereby demonstrating promising anticancer activity in several preclinical models of pancreatic cancer, including primary cultures^[24]^. In the current study, we assessed the antitumor activities of the most potent compounds 10k and 10l in our NSCLC models. These compounds demonstrated excellent antitumor activity, significantly reducing p-FAK levels, consistent with *in silico* studies suggesting these compounds as promising candidates for developing new inhibitors of FAK and its downstream oncogenic pathways. Specifically, they strongly inhibited cell proliferation, survival, and migration, while increasing apoptosis in various NSCLC cell lines with different EGFR mutations and EGFR-TKI resistance profiles. In particular, compounds 10k or 10l induced apoptosis and decreased the levels of the anti-apoptotic gene *BCL2 in vitro* and 10k decreased p-Akt, inducing activation of caspase-3, a key effector of apoptosis *in vivo*. Interestingly, it was previously shown that overexpression of *BCL2* family (such as *BCL2* and *BCL-XL*) in the apoptotic signaling pathway, blocked apoptosis and reduced the sensitivity of cells to EGFR-TKI-induced apoptosis^[44-46]^. Enhancing the sensitivity of tumor cells to apoptosis by treatment with BCL2 inhibitors, such as ABT737, or other apoptosis inducers can effectively overcome EGFR-TKI resistance^[45,47]^. Indeed, the combination of lapatinib (a dual EGFR and HER2 inhibitor) with BCL2/BCL-XL inhibitors has been shown to reverse matrix-induced lapatinib resistance^[48]^, warranting future studies to evaluate the potential synergy between 10k or 10l and lapatinib.

We further investigated the roles of 10k and 10l in surmounting EGFR-TKI resistance, in combination with EGFR-TKIs. Through SRB assays, colony survival assays, and spheroid formation, we found that when combining them with EGFR-TKI, they displayed an enhanced inhibition of tumor cell proliferation when compared with single drugs in the resistant cells. Consistently, 10k and 10l exhibited excellent CIs when combined with Afatinib or Osimertinib. In addition, BCL2 was highly expressed in drug-resistant cells, and when used in combination with EGFR-TKIs, 10k or 10l synergistically induced apoptosis in these drug resistant cells. Remarkably, 10k was also able to significantly suppress tumor growth *in vivo* and enhance the Osimertinib sensitivity of TKI-resistant xenograft models. Thus, we demonstrated that 10k and 10l are promising FAK inhibitors in NSCLC that resensitize EGFR-TK-resistant tumor to Afatinib or Osimertinib treatment. However, our current and previous studies^[24]^ demonstrated that they inhibited the activity of other kinases, including the EPH and STAT families, which have also been associated with acquired resistance to EGFR-TKIs^[49,50]^. Therefore, the superior synergistic activity of 10k and 10l in combination with EGFR-TKIs might not have depended solely on inhibition of FAK activity.

Our pharmacological study has several limitations. First, we did not investigate the precise mechanisms by which FAK overexpression drives the acquisition of resistance to EGFR-TKIs. To gain preliminary insight into downstream mechanisms, we assessed p-Akt inhibition, induction of apoptosis (via caspase-3 activity), and suppression of cell proliferation (via Ki-67 staining) following 10k treatment in our *in vivo* models. A deeper understanding of FAK-mediated, EGFR-coupled signaling cascades will be essential for developing strategies to overcome acquired EGFR-TKI resistance in NSCLC.

Second, although we demonstrated that our compounds can overcome EGFR-TKI resistance *in vitro*, we did not perform comprehensive analyses of treatment-induced changes in gene expression or signaling pathways. Future studies incorporating RNA sequencing and (phospho)-proteomics to map mechanistic pathways will be important to elucidate FAK regulation and clarify how its inhibition reverses resistance, ultimately guiding the optimization of therapeutic strategies for NSCLC patients.

Despite these limitations, our study has several strengths. For instance, by integrating data from diverse sources - clinical patient samples, public databases, and well-established *in vitro* models - we offer a robust multi-faceted analysis of the role of FAK in EGFR-TKI resistance. Additionally, the promising *in vivo* antitumor activity observed with our 10k compound suggests its potential as a viable therapeutic agent, laying a strong foundation for future translational investigations.

In conclusion, our results provide strong evidence that elevated FAK levels are a key driver of acquired EGFR-TKI resistance and can serve as a biomarker for poor prognosis in NSCLC patients treated with EGFR-TKIs. This underscores the importance of developing novel FAK inhibitors. The novel FAK inhibitors 10k or 10l, significantly reduced p-FAK levels and exhibited potent antitumor activity in both EGFR-TKI-sensitive and -resistant NSCLC cells harboring activating EGFR mutations, *in vitro* and *in vivo*. Additionally, 10k and 10l significantly enhanced the sensitivity of EGFR-TKI-resistant cells to EGFR-TKIs. These findings reveal a promising therapeutic avenue for patients who develop resistance to EGFR-TKIs, suggesting that compounds such as 10k and 10l could be used either as standalone treatments or in combination with EGFR-TKI therapy to enhance clinical outcomes.

## DECLARATIONS

### Availability of data and materials

The raw data supporting the findings of this study are available within this article and its Supplementary Materials. Further data are available from the corresponding author upon reasonable request.

### Copyright

© The Author(s) 2025.
