# Supplementary material for: Novel FAK inhibitors suppress tumor growth and reverse EGFR-TKI resistance in non-small cell lung cancer
Source: Cancer Drug Resist. 2025 Nov 5;8:57. doi: 10.20517/cdr.2025.139 (PMC12635985; doi:10.20517/cdr.2025.139)
Supplement: Supplementary file 1 [file cdr-8-57-SupplementaryMaterials.pdf]

Supplementary Materials

Novel FAK inhibitors suppress tumor growth and reverse EGFR-TKI resistance in non-small cell lung cancer

Geng Xu[1,2], Camilla Pecoraro[1,3], Mahrou Vahabi[1], Dongmei Deng[2], Andrea Cavazzoni[4], Hamid Fiuji[5], Costanza Anna Maria Lagrasta[4], Stella M. Cascioferro[3], Marcello Tiseo[4,6], Daniela Carbone[3], Amir Avan[5,7], Paolo A. Zucali[8,9], Yehuda G. Assaraf[10], Godefridus J. Peters[1,11], Patrizia Diana[3], Elisa Giovannetti[1,12]

[1]Department of Medical Oncology, Cancer Center Amsterdam, Amsterdam UMC, Vrije Universiteit University, Amsterdam 1081 HV, the Netherlands.

[2]Department of Preventive Dentistry, Academic Centre for Dentistry Amsterdam (ACTA), University of Amsterdam and Vrije Universiteit Amsterdam, Amsterdam 1081 LA, the Netherlands.

[3]Department of Biological, Chemical, and Pharmaceutical Sciences and Technologies (STEBICEF), University of Palermo, Palermo 90128, Italy.

[4]Department of Medicine and Surgery, University of Parma, Parma 43126, Italy.

[5]Metabolic Syndrome Research Center, Mashhad University of Medical Sciences, Mashhad 9177899191, Iran.

[6]Department of Oncology and Hematology, Medical Oncology Unit, University Hospital of Parma, Parma 43126, Italy.

[7]Faculty of Health, School of Biomedical Sciences, Queensland University of Technology (QUT), Brisbane 4059, Australia.

[8]Department of Oncology, IRCCS Humanitas Research Hospital, Rozzano 20089, Italy.

[9]Department of Biomedical Sciences, Humanitas University, Pieve Emanuele 20072, Italy.

[10]The Fred Wyszkowski Cancer Research Laboratory, Faculty of Biology, Technion-Israel Institute of Technology, Haifa 3200003, Israel.

[11]Department of Biochemistry, Medical University of Gdansk, Gdansk 80-211, Poland.

[12]Cancer Pharmacology Lab, Fondazione Pisana per la Scienza, San Giuliano 56017, Italy.

Correspondence to: Dr. Elisa Giovannetti, Department of Medical Oncology, Cancer Center Amsterdam, Amsterdam UMC, Vrije Universiteit University, Amsterdam 1081 HV, the Netherlands. E-mail: e.giovannetti@amsterdamumc.nl

Supplementary Methods

Cell cultures, drugs and reagents

The human NSCLC cell lines PC9 and H1975, obtained from the American Type Culture Collection (ATCC), were cultured in RPMI-1640 medium supplemented with 10% newborn calf serum for nutrient support, 100 U/mL penicillin, and 100 μg/mL streptomycin (Lonza, Basel, Switzerland). Cells were maintained in a humidified incubator at 37 °C with 5% CO_2. All cell lines were genotyped and routinely tested for Mycoplasma contamination.

The synthesis protocols and detailed procedures for compounds 10k and 10l were provided in our previous study[1]. Afatinib and Osimertinib were purchased from MedChemExpress (MCE, Monmouth Junction, NJ, USA) and prepared according to the manufacturer's specifications. All other reagents, unless otherwise stated, were obtained from Sigma-Aldrich (St. Louis, MO, USA).

Generation of drug-resistant tumor cells

PC9 Afatinib-resistant (AR) and H1975 Osimertinib-resistant (OR) sublines were generated using a stepwise selection protocol. In brief, the initial drug treatment of parental cells with EGFR-TKI concentrations based on the half-maximal inhibitory concentration (IC_{50}) was continued for at least 2 weeks until an 80% confluency was achieved. Once resistance began to emerge, the concentration of the drug was doubled, a process repeated until cells acquired stable resistance, requiring 6-8 months of drug selection. The cell IC_{50} values were periodically assessed, and for following experimental tests, cells were cultured without drugs for at least 2 weeks.

Cell mutations and copy number analyses

Mutation analyses were conducted as part of routine molecular diagnostics for EGFR mutation assessment at Amsterdam University Medical Centers, VU University, Amsterdam, The Netherlands. This included Sanger sequencing, high-resolution melting analysis, and a multiplexed cancer panel for targeted resequencing[2]. These assays are designed to detect deletions or insertions in EGFR exons 19 and 20, as well as hotspot mutations in EGFR exons 18 through 21. In addition, in situ hybridization (ISH) was performed to determine the cMET gene copy number using the MET/CEP7 Dual Color Probe (Ventana). The procedure followed the

manufacturer's guidelines for detecting cMET gene amplification, a molecular mechanism implicated in resistance to EGFR-TKIs[3].

Evaluation of cell growth proliferation using the sulforhodamine B (SRB) assay

Cells were plated at a density of 5,000 cells/well (in 100 μL growth medium) in a 96-well plate and incubated overnight. After treating the cells with the drugs for 72 h, 25 μL/well of 50% trichloroacetic acid (TCA) were added and the cells were fixed at 4 °C for 1 h. TCA was then aspirated, and cells were gently washed several times, and allowed to air-dry at room temperature. Thereafter, 50 μL/well of 0.4% mass fraction of SRB dye solution was added, and left at room temperature for 30 min. The dye solution was then discarded and a washing buffer (1% acetic acid by volume) was added in order to remove any remaining dye solution from the walls of the wells while fixed cells were allowed to air-dry. Finally, 150 μL/well of SRB dissolved solution (10 mM Tris base solution) was added at room temperature for 5 min and the absorbance was measured at a wavelength of 490 nm using a BioTek plate reader (BioTek Instruments Inc., Winooski, VT, USA).

Evaluation of pharmacological interaction

The combination index (CI) was calculated to compare the cell growth inhibition of the combination with each individual drug. Data analysis was performed using CalcuSyn software (Biosoft, Oxford, UK).

Colony formation assay

Cells were seeded at a density of 1,000 cells/well in a 6-well plate. After 24 h of incubation, the cells were treated with the drugs and the culture medium was replaced every 3 days. After 10 days, the culture medium was removed, and the cells were washed with PBS. The cells were then fixed with 100% ethanol for 15 min and allowed to air dry. Cells were then stained with a solution of 0.5% crystal violet for 30 min. After removing the staining solution, the colonies were washed with water, air-dried, and photographed using GelCount (Oxford Optronix, Oxford, United Kingdom). Only clones with > 50 cells were scored. Plating efficiency (PE) and cell survival fractions (SF) were calculated as described previously[4].

Analysis of antitumor activity in multicellular spheroids

Cells were seeded at a density of 3,000 cells/per well in Ultra-Low Attachment (ULA) 96-well plates (Costar, Washington, DC, USA). After centrifugation, the plates were incubated in a cell culture incubator until spheroids formed. The spheroids were then treated, and the medium was refreshed every 2 days. Imaging was performed using a Leica DMI300B microscope (Leica Microsystems, Eindhoven, Netherlands) at a magnification of 5X. After 4 days, the culture medium was removed, and spheroids were dissociated with trypsin. Following cell spheroid dissociation, neutralization with serum, and centrifugation, the dissociated cells were seeded in regular 96-well plates. After 5 days, cell proliferation was evaluated using resazurin assay[5]. Specifically, 10 μL resazurin solution was added in each well, and after 1 h, fluorescence was determined using a BioTek plate reader.

Analysis of cell migration by wound-healing assay

The wound healing assay was performed to assess cell migration capability, as described previously[6]. PC9, PC9AR, H1975, and H1975OR cells were seeded at a density of 5×10^4 cells/well in a 96-well flat-bottom plate with 0.2 mL growth medium. After pre-incubation at 37 °C, 5% CO_2, and 100% humidity for 24 h, a uniform scratch was created using a scraper. The culture medium was then removed, and the detached cells were washed away with PBS. Subsequently, medium containing different compounds was added. Wound closure was monitored using a phase-contrast microscope (Universal Grab 6.3, Digital Cell Imaging Labs, Keerbergen, Belgium) integrated into a Leica DMI300B system (Leica Microsystems), and images were captured immediately at 0, 4, 8, and 20 h. Data analysis was performed using Scratch Assay 6.2 software (Digital Cell Imaging Labs).

Enzyme-Linked Immunosorbent Assay (ELISA) for phosphorylated FAK

The levels of phosphorylated FAK (p-FAK) protein were measured using a specific ELISA (Thermo Fisher, KHO0441)[7]. Tumor tissues were homogenized in ice-cold lysis buffer containing protease and phosphatase inhibitors, while cells were directly lysed in ice-cold lysis buffer. The lysates were clarified by centrifugation at 4 °C, and the supernatants were collected. Protein concentrations were determined using a BCA assay (Thermo Scientific™ Pierce™, 23225) and samples were diluted

according to the ELISA kit instructions. 50 μL of standard, control, or sample was added to each well and incubated for 2 h at room temperature. Then, add 50 μL of FAK [pY397] Detection Antibody solution into each well and incubated for 3 h at room temperature. After washing four times with PBS, the anti Rabbit IgG HRP solution was added to each well and incubated for 30 min at room temperature. After washing four times with PBS, 100 μL of substrate solution was added to each well, and the reaction was terminated after 30 min by adding the stop solution. The absorbance at 450 nm was measured using a BioTek plate reader.

Patient tissues and immunohistochemistry (IHC)

Between 2006 and 2020, NSCLC tissue samples were collected from patients treated at Vrije Universiteit Medical Center (Amsterdam, The Netherlands), Humanitas Clinical Institute (Rozzano, Milan, Italy), and Parma University Hospital (Parma, Italy), according to protocols approved by the local Ethics committees. According to the revised international cancer staging system, all cases were clinically and pathologically confirmed as NSCLC. Patients selected for the current study received EGFR-TKI treatment for advanced EGFR-mutated NSCLC. All participants provided a written informed consent at the time of recruitment.

IHC was performed to detect the expression of p-FAK in patient tissues and ki67 in mice specimens, respectively. Briefly, tissues were subjected to deparaffinization, hydration, and antigen retrieval. The samples were incubated overnight with anti-p-FAK antibodies (Thermo Fisher, clone 31H5L17) or Ki67 (Thermo Fisher, MA5-14520) at 4 °C, followed by secondary incubation and color development using 3,3'-Diaminobenzidine. Based on the method previously described[8], staining scores for the protein-FAK were blindly assessed based on the product of staining intensity levels (representing negative, weak, moderate, or strong staining with 0, 1, 2, or 3, respectively) and positive staining percentages (representing 0% to 5%, 6% to 25%, 26% to 50%, 51% to 75%, or 76% to 100% of positive areas with 0, 2, 3, or 4, respectively). The median was chosen as a cut-off, and samples were defined as "high p-FAK" when the staining score was > the median, and "low p-FAK" when the staining score was ≤ the median. The Ki67 expression was quantified with ImageJ Color Deconvolution (H DAB) using tumor tissues from three mice, with three 20× images per tumor. Significance was determined using an unpaired, two-tailed Student's *t* test.

Analysis of apoptosis *in vitro*

Apoptosis was evaluated using methods described in previous studies[1,6]. Regarding the Annexin V/PI (Thermo Fisher Scientific Inc) staining assay which was performed to assess cell death and apoptosis induction, cells were seeded at a density of 1 × 10^4 cells/well in a 96-well plate and incubated overnight. Then the cells were treated with 10k, 10l, Afatinib, Osimertinib and their combination for 2 days. Following removal of the supernatant and washing with PBS, cells were incubated with binding buffer containing Annexin V and PI for 10 min at room temperature. Fluorescence values were measured using a BioTek plate reader, at excitation/emission wavelengths of 485/535 nm and 536/617 nm, respectively.

For the direct detection of Annexin V, images of cells were obtained with a Leica DM2500 fluorescence microscope (Leica Microsystems) using a fluorescein isothiocyanate (FITC) filter set to visualize fluorophores emitting green light.

Analysis of p-Akt and apoptosis *in vivo*

Tumor levels of phospho-AKT were determined in stored supernatants from homogenized tumor xenografts H1975OR tissues of using a specific ELISA assay (Invitrogen™ Human AKT (Phospho) [pS473] ELISA Kit, Thermo Fisher Scientific, Waltham, MA, USA) for AKT phosphorylation at serine-473 (AKT [pS473]), as described previously[9]. Briefly, tissue extracts were prepared following the manufacturer's instructions, then incubated on ice and centrifuged to obtain the supernatant. Phospho-Akt levels were determined with rabbit anti-phospho-Akt and HRP-conjugated secondary antibodies, and absorbance measured at 450 nm. Concentrations were calculated using a standard curve, normalized to total protein content assessed by the BCA assay (Thermo Scientific™ Pierce™), as described above, and expressed relative to control mice.

To assess whether caspase-3, a key enzyme in the effector phase of apoptosis, was also involved in the downstream effects of our novel FAK inhibitor, its activity was measured using a specific spectrofluorimetric activity assay (Human Active Caspase-3 Immunoassay Quantikine ELISA, R&D Systems, Inc., Minneapolis, MN). Briefly, frozen tumor specimens were lysed and processed according to the manufacturer's protocol. Absorbance was measured at 450 nm, with background

correction at 540 nm. Relative caspase-3 activity was normalized to total protein content and expressed relative to control mice, as described above.

Analysis of gene expression by Reverse Transcription Quantitative Polymerase Chain Reaction (RT-qPCR) and PCR array

Total RNA was extracted from cells using TRI REAGENT-LS (Invitrogen, Carlsbad, CA), following the manufacturer's protocol, with minor modifications[10]. The RNA concentration and purity were assessed at 260 to 280 nm using a NanoDrop-1000 spectrophotometer (NanoDrop Technologies, Wilmington, DE, USA). Reverse transcription of 0.5 μg of RNA was performed using the DyNAmo cDNA Synthesis Kit (Thermo Scientific, Vantaa, Finland).

For quantitative PCR, specific TaqMan® primers and probes for Matrix Metalloproteinase-9 (MMP9), B-cell Lymphoma 2 (BCL2), and FAK were used, as described previously[1,11]. The cDNA was amplified using an ABI-PRISM 7500 instrument (Applied Biosystems, Foster City, CA, USA). Amplification data were normalized to beta-actin, and quantification of gene expression was performed using standard curves obtained with dilutions of cDNA from Quantitative-PCR Human-Reference Total-RNA (Stratagene, La Jolla, CA). Data were also analyzed according to the $2^{-\Delta\Delta Ct}$ method.

Gene expression differences of pivotal oncogenic kinases in H1975 and H1975OR were evaluated using a RT2 Profiler™ PCR Arrays (Qiagen, Hilden, Germany), according to the manufacturers' protocol. This array includes 84 key kinases that form the molecular machinery driving the modulation of oncogenic pathways. Equal amounts of total RNA (0.5 μg) were reverse transcribed into cDNA using the RT² First Strand Kit (Qiagen). Real-time PCR was then performed with SYBR Green Mastermix and the Human Kinases RT² Profiler PCR Array (Qiagen) on an ABI PRISM™ 3100 (Applied Biosystems, Foster City, CA) machine, following the manufacturer's instructions. Fold changes in mRNA expression were analyzed using SABiosciences' web portal software, with significance assessed by Student's *t*-test. A P-value < 0.05 and a fold change > 2.0 were considered statistically significant.

Evaluation of kinases activity profiles

A PamChip® array (PamGene International, 's-Hertogenbosch, The Netherlands) was used to test the change in tyrosine kinase activities in resistant cells. This chip contains 136 phosphorylation sites of 144 peptides each consisting of 15 amino-acids. The 13 amino-carboxylic acids (R-COOH) of each peptide correspond to known or putative phosphorylation sites of tyrosine kinases. The experiments were performed in biological triplicates, as described previously[1].

Cells were cultured in 25 cm^2 flasks at 37 °C with 5% CO_2 until reaching 75% confluence. Cells were detached, and 1×10^6 cells per mL from each replicate were collected. Cell lysis was performed by adding 100 μL of M-PER lysis buffer (Thermo Scientific, Rockford, IL, US) per million cells, which included Halt protease and phosphatase inhibitors at a 1:100 dilution. The lysis process occurred over 15 min at 4 °C, and lysates were centrifuged at 16,000 g for 15 min at 4 °C. Protein concentrations were measured using the Bradford method (Biorad, Hercules, CA, USA), and samples were adjusted to 10 μg protein per array for PamChip preparation. These samples were combined with the MasterMix PamGene reagent kit, containing PK buffer, BSA, PTK additive, DTT, protease and phosphatase inhibitors, PY-20-FITC antibody (Exalpha, Maynard, MA, USA), and ATP. Sample mixtures were loaded onto the chip following a blocking step with 2% BSA and PK buffer washes on the PamStation®12. Each mix was loaded at least in duplicate, incubated at 30 °C, and cycled through the array for 60 cycles to ensure even distribution. Fluorescence intensities were monitored in real-time with a 12-bit CCD camera, and end-point intensities were analyzed using the using Bionavigator software 5.1 (Pamgene International). Background normalization was performed, adjusting values such that the negative control equaled to zero. Differential analysis was carried out using Student's *t*-test in R (version 3.6.1), and the resulting *p*-values were corrected for false discovery rate (FDR). Peptides with an FDR < 0.01 were considered statistically significant.

Bioinformatics analyses

Genes with a fold change greater than 2 and a *P*-value < 0.05 were selected for further bioinformatics analysis to identify significant changes in gene expression. Protein-protein interaction (PPI) was performed using the STRING database to

explore interactions among these genes[12], with visualization conducted in Cytoscape (version 3.5.0). Additionally, Kyoto Encyclopedia of Genes and Genomes (KEGG)[13] pathway enrichment analysis was carried out using the “enrichR” package in R, providing insights into the biological pathways associated with these genes, and results were visualized with the “ggplot2” package[14].

RNA sequencing data and patient clinical information were retrieved from The Cancer Genome Atlas (TCGA) via the UCSC Xena dataset platform (http://xena.ucsc.edu/welcome-to-ucsc-xena/). The raw gene count data were normalized using the DESeq2 package in R, enabling accurate comparison of gene expression levels. To evaluate differences in gene expression between normal and tumor tissues, the Wilcoxon Mann-Whitney test was applied, ensuring robust statistical assessment to identify and confirm key genes associated with tumor biology.

Docking analysis

The ligands 10k and 10l for docking were prepared using the same tools reported previously[15], through the LigPrep tool, available in the Maestro Suite, Schrodinger software[16]. All possible tautomers and stereoisomers were generated for a pH of 7.0 ± 0.4, using the Epik ionization method[16]. Consequently, the integrated Optimized Potentials for Liquid Simulations (OPLS) 2005 force field was used to minimize the energy status of the ligands[17]. The high-resolution crystal structure of FAK (PDB ID: 6YOJ) was downloaded from the Protein Data Bank[18] and prepared using the Protein Preparation Wizard, in the Schrodinger software[16]. This included assigning bond orders, adding hydrogen atoms, deleting water molecules, and optimizing the H-bond network, followed by restrained energy minimization (RMSD 0.3 Å) using the OPLS 2005 force field[17]. Molecular Docking studies were executed and scored using the Glide module, with receptor grids centered on the original ligands. The generated 3D conformers were docked into the receptor model using the Extra Precision (XP) mode as the scoring function. Induced Fit Docking simulations were performed using the IFD application, accounted for both ligand and receptor flexibility[19,20], and molecules were ranked based on the docking score.

Evaluation of the antitumor activity

We follow The ARRIVE Guidelines 2.0[21]: updated guidelines for reporting animal research *in vivo* study.

In vivo studies were carried out using four- week- old female nude mice with an average weight of 21 g (ranging from 20 to 22 g upon arrival), and their weight measurements during the study can be found in Supplemental Figure 12.

We selected female mice based on (1) the clinical enrichment of EGFR mutations in women, which makes a female host an appropriate baseline for xenografts, and (2) practical considerations that female mice exhibit lower aggression and more stable subcutaneous tumor take/growth, improving data consistency and aligning with the “replacement, refinement, and reduction (the “3Rs”) of animal use” by minimizing variability and animal use.

These mice were sourced from the Charles River Laboratories (Calco, Italy) and Pasteur Institute (Tehran, Iran). The experimental protocol received approval from Local Ethical Committees of the Mashhad University of Medical Science with IR.MUMS.MEDICAL.REC.1403.294 reference number, and University of Parma, in accordance with the institutional guidelines that are in compliance with national (DL116/92) and international (86/609/CEE) laws and policies.

Additionally, our study design and reporting adhered to the ARRIVE guidelines, with a completed ARRIVE checklist provided as supplemental material to indicate where each item is addressed in the manuscript.

To specifically reduce the influence of subjective bias during the allocation of animals to treatment groups, we implemented a randomization process—matching animals based on both tumor size and body weight—and ensured that pathologists assessed the results in a blinded manner. The mice were kept in groups of three per cage under pathogen-free (SPF) conditions, with a standard light/dark cycle, controlled temperature, and unrestricted access to food, water, and environmental enrichment. Welfare assessments and any necessary interventions were performed both prior to and during the experiment.

NSCLC models (n = 6 tumors per treatment group) were developed by injecting 2.5 × 10^6 H1975 or H1975OR cells subcutaneously into the right flank of each mouse (1). To alleviate postoperative discomfort, temgesic was given subcutaneously at doses between 0.05 and 0.1 mg/kg, as its efficacy as an analgesic has been previously confirmed[22]. Once the tumor volumes reached an average of approximately 100 mm^3, the animals were randomly divided into five groups (with six mice per group) as follows: (1) untreated controls; (2) a group treated solely with 10k at 20 mg/kg on 3 days/week for two weeks (with the formulation prepared at 5 mg/mL in PBS, delivering 100 μL intraperitoneally per 25 g mouse); (3) a group treated solely with Osimertinib at 3 mg/kg, 3 days/week for two weeks (formulated at 0.75 mg/mL in PEG400); (4) a group receiving a combined treatment of 10k and Osimertinib at the aforementioned doses for two weeks; and (5) a group treated with Defactinib, solubilized in PEG400 at 50 mg/kg for 3 days/week for two weeks (with a formulation concentration of 12.5 mg/mL in PEG400). The dose administered was based on the results of preliminary experiments and data from our previous studies[1,23]. Tumor xenograft measurements were performed according to the methodology described by Cavazzoni *et al.*[23]. All treatments were administered in the morning within the animal facility laboratory, and each experimental group was treated and evaluated in the same sequential order. Local anesthesia for the mice was provided by applying lidocaine to the skin. After the first week, mice were weighted every three days. At the end of the experiment, tumors were harvested, weighed, and stored for further studies.

Our previous study showed that the NSCLC cell line typically exhibit high tumorigenic potential in subcutaneous tumor models[23]. Moreover, standardized cell culture and injection procedures can significantly reduce the variability in tumor volume, thereby ensuring a relatively uniform growth curve within each group. When the anticipated interventions—such as gene regulation or pharmacological treatments—are expected to induce substantial biological effects, a group size of six mice generally provides sufficient statistical power to detect significant intergroup differences[24]. Additionally, in accordance with the 3R principles (Replacement, Reduction, and Refinement), employing a smaller sample size, while still ensuring the reliability of experimental results, effectively minimizes animal usage and

consequently reduces the ethical burden associated with animal welfare. Taken together, using six mice per group not only guarantees reproducibility in tumor take rate and tumor volume measurements but also meets the requirements for statistical robustness and animal ethics.

With respect to how our experimental methods and results inform the replacement, refinement, and reduction (the "3Rs") of animal use, our protocol stated that developing a mouse model from NSCLC cell cultures is vital for enhancing our understanding of the biology of this devastating cancer. Moreover, such models facilitate preclinical studies for novel therapeutic strategies, serving as a platform to evaluate drugs that could potentially benefit patients afflicted by this lethal disease. To date, many clinical trials have been launched without solid preclinical evidence or by relying on conventional models that do not accurately represent the original tumor. This gap in appropriate preclinical research may contribute to the disappointing outcomes in clinical trials and the subsequent lack of improvement in patient survival rates. Given the urgent need to improve NSCLC treatment, the use of animals—and the moderate to severe distress they might experience—is considered acceptable. We are confident that our research will enable the discovery of innovative, effective therapies for patients battling this aggressive cancer. Furthermore, our rigorously designed and analyzed animal studies are expected to accelerate the development and adoption of cutting-edge models and tools based on the latest scientific and technological advances, ultimately reducing animal usage in future research.

Statistical analysis

Experiments were performed in triplicates and repeated at least twice. The percentages of cell migration were calculated taking into account at least six scratches. Data analysis was performed using Excel, SPSS software (version 19.0, SPSS, Chicago, IL, USA), R studio (https://www.R-project.org/) and Graphpad Prism version 9.0 (Intuitive Software for Science, Boston, MA, USA), and graphs were generated using Graphpad. Data are shown as mean ± standard deviation. Statistical significance was assessed using the two-tailed Student's *t*-test, or one-way ANOVA with multiple comparisons. Normality was assessed using the Shapiro–Wilk test. Survival analysis was evaluated by Kaplan-Meier method and p-value was

calculated by logrank (Mantel-Cox test). The association between FAK expression and clinicopathological features of lung cancer was performed by the Spearman rank correlation coefficient. The cut-off level of statistical significance was $P < 0.05$.

Supplementary Table 1. Clinicopathological characteristics of lung cancer patients and correlation with overall survival (OS)

**Characteristic**	**Patients**	**OS months (95%CI)**	***P*-value**
Gender	n (%)		0.448
Male	21(70)	6.7[3.9-9.5]	
Female	9(30)	7.5[3.9-10.9]	
Age (years)			0.301
≤65	22(73)	5.7[4.1-7.3]	
>65	8(27)	7.7[3.3-12.1]	
Stage			0.007**
I/II	8(27)	11.4[5.2-17.5]	
III/IV	22(73)	5.3[3.6-6.9]	
PS			0.041*
0/1	27(90)	5.8[3.9-7.6]	
2	3(10)	2[-2.3-6.3]	
smoking			0.601
yes	25(83)	7.2[4.8-9.7]	
no	5(17)	5.2[1.2-9.2]	
Pathological type			0.677
SPINO	7(23)	6[2.1-9.9]	
ADC	21(70)	7.2[4.4-10]	
NAS	2(7)		

This table summarizes the clinicopathological features of lung cancer patients, including age, tumor stage, histological subtype, and other relevant factors. Statistics was performed by the Spearman rank correlation coefficient. ADC: Adenocarcinoma; NAS: not otherwise specified; PS: performance status; SPINO: squamous cell spinocarcinoma. Statistical significance: *P*-values was set as follows: $^{**}P < 0.01$.

Supplementary Table 2. The half-maximal inhibitory concentrations (IC_{50}) of the compounds in NSCLC cell lines, demonstrating their potency and efficacy in inhibiting cell proliferation

Cell lines	10k (μM)	10l (μM)	Defactinib (μM)
PC9	7.6±1.9	3.1±0.27	4.3±1.7
PC9AR	9.1±0.4	3.5±1.29	4.0±0.1
H1975	5.9±1.2	3.1±1.1	1.1±0.2
H1975OR	5.8±0.1	2.6±0.9	2.0±0.2

Data are derived from dose-response SRB experiments conducted in triplicates and are presented as mean ± SD.

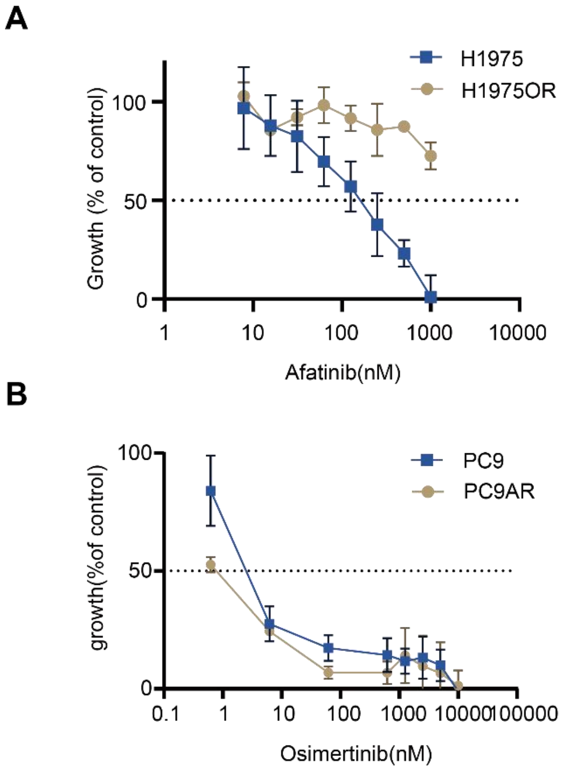

Supplementary Figure 1. Analysis of cross resistance in EGFR-TKIs resistant cell lines. H1975/H1975OR (A) and PC9/PC9AR (B) cells were treated with the increasing amounts of indicated drugs (Afatinib or Osimertinib) for 72 h. The growth rate was measured by SRB assay.

A

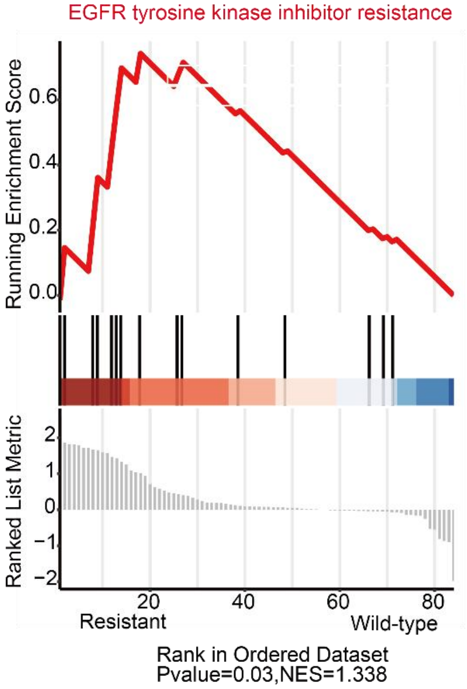

B

Gene ID	Protein name	Log2 Fold change	-Log10(Padj)
PDGFRB	Platelet-derived growth factor receptor beta	1.67	5.52
SRC	Proto-oncogene tyrosine-protein kinase Src	1.86	5.95
AXL	Tyrosine protein kinase receptor UFO	1.01	4.34
FGFR3	Fibroblast growth factor receptor 3	1.33	5.31
MET	Hepatocyte growth factor receptor	1.47	5.67
EGFR	Epidermal growth factor receptor	1.66	5.59
FGFR2	Fibroblast growth factor receptor 2	1.44	5.59

The fold change is based on H1975OR/H1975WT

Supplementary Figure 2. Gene Set Enrichment Analysis (GSEA) highlighting EGFR-TKI resistance pathways in H1975OR cells compared to H1975 cells. (A) GSEA plot depicting the enrichment of resistance-related pathways; (B) List of specific genes enriched in EGFR-TKI resistance pathways, demonstrating their upregulation in H1975OR cells. Notably, these are the genes that also emerged in the PPI analysis in Figure 1E.

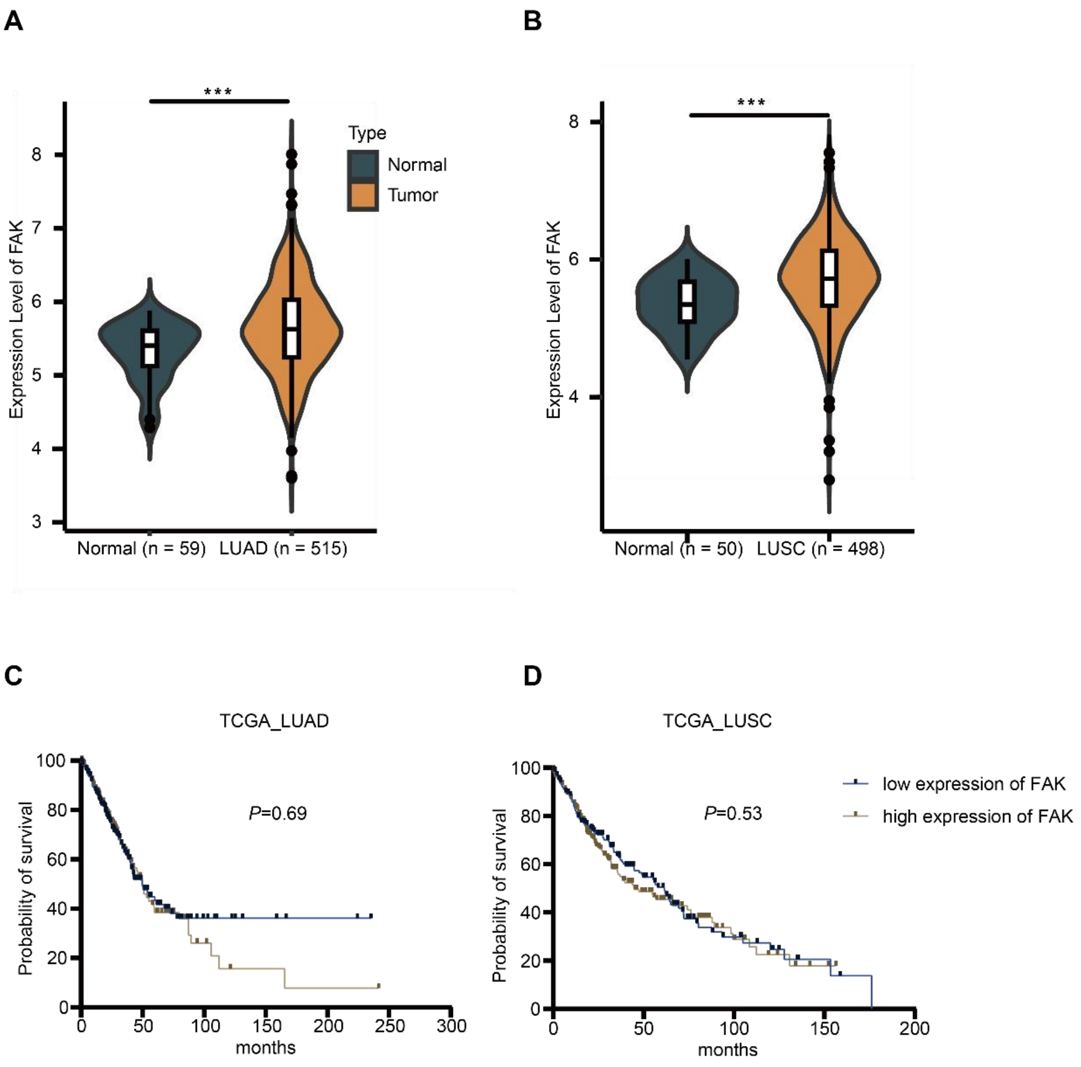

Supplementary Figure 3. Gene expression of FAK in lung cancer tumor and normal tissues was analyzed using the TCGA dataset. Expression analysis of FAK mRNA in TCGA-LUAD (A) and TCGA-LUSC (B) revealed significantly higher levels in tumor tissues compared to normal lung tissues; However, survival analysis of TCGA-LUAD (C) and TCGA-LUSC (D) patients comparing low versus high FAK expression showed no significant differences in overall survival. Statistical significance: *P*-values were set as follows: $^{***}P < 0.001$.

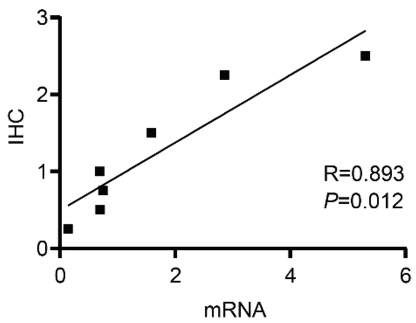

Supplementary Figure 4. Correlation analysis of FAK mRNA expression and immunohistochemistry (IHC) scores for phosphorylated FAK (P-FAK) in tissue samples from 7 NSCLC patients. The analysis highlights the potential relationship between transcriptional and protein activation levels of FAK in these patient-derived samples.

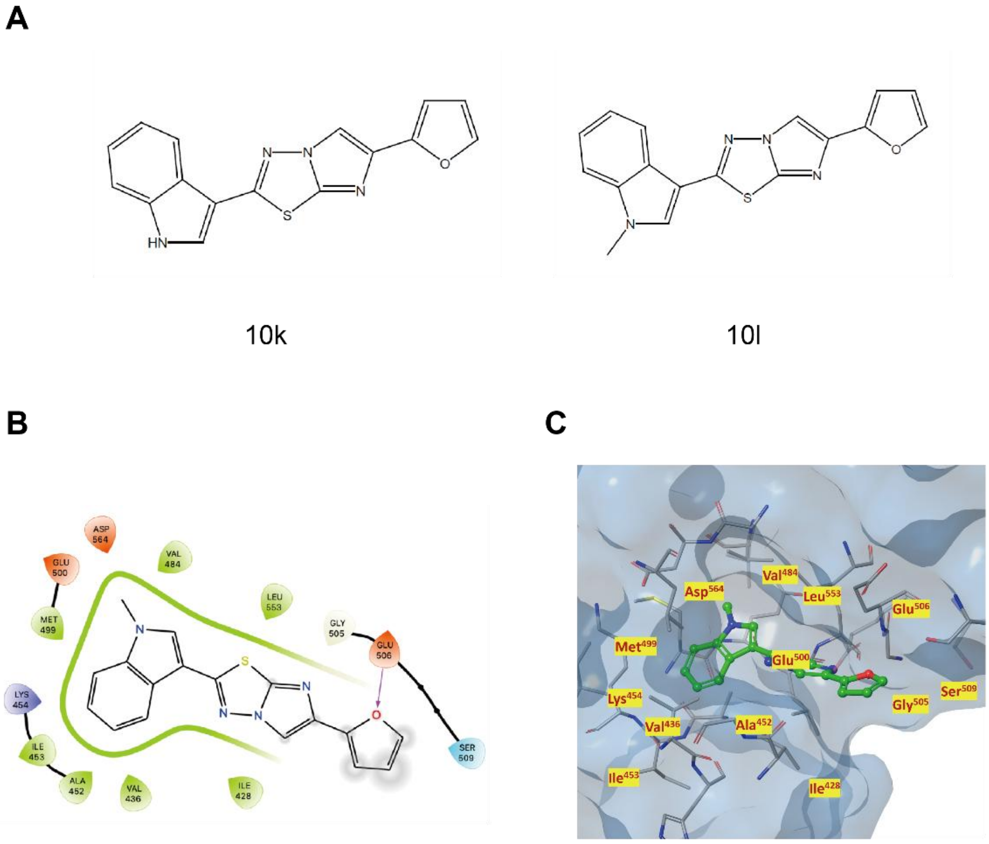

Supplementary Figure 5. Structure-interaction of 10l with FAK. (A) Chemical structures of 10k and 10l; (B) 2D ligand interaction diagram of compound 10l, showing interactions within the binding pocket. Afa, Afatinib; Osi, Osimertinib; (C) 3D complex of the FAK catalytic domain (PDB code 6YOJ) with compound 10l. Nitrogen, oxygen, and sulfur atoms are depicted in blue, red, and yellow, respectively.

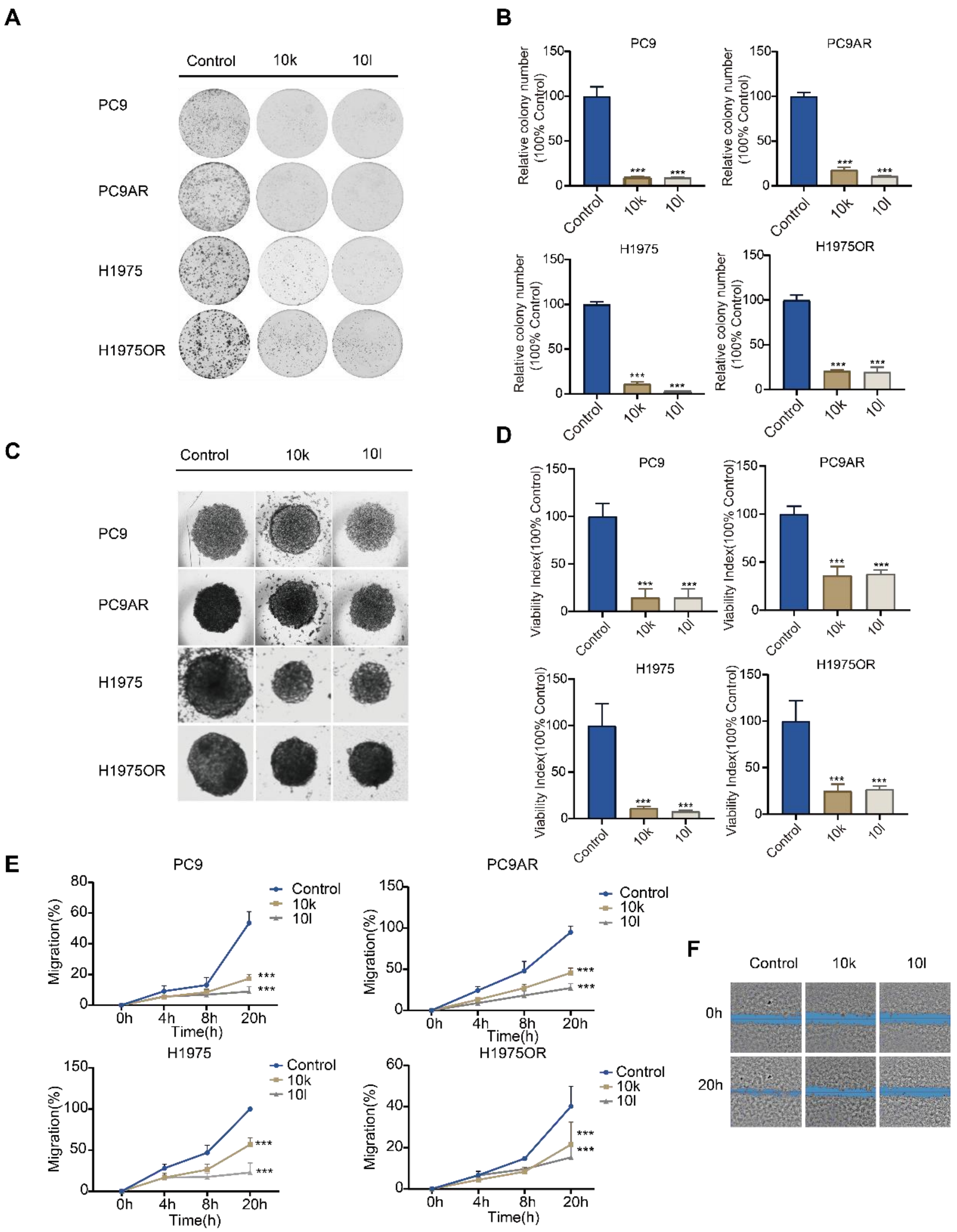

Supplementary Figure 6. The new FAK inhibitors 10k and 10l inhibited proliferation and migration of EGFR-TKI-sensitive and -resistant NSCLC cells. (A and B) NSCLC cells were seeded in 6-well plates at a density of 1 × 10^3 cells/well and treated with each compound for 10 days. Representative images of the colony formation assay (left panels) and quantitative analysis of colony numbers (right panels) are shown. PC9 cells or PC9AR cells: treated with 10k or 10l at 0.5 × IC_{50} (PC9AR cells), H1975 cells or H1975OR cells: treated with 10k or 10l at 0.5 × IC_{50} (H1975OR cells); (C and D) spheroids were treated with 10k or 10l for 4 days.

Representative images of the spheroids (left panels) and their viability, assessed using a resazurin assay, are shown (right panels). PC9 cells or PC9AR cells: treated with 10k or 10l at 3× IC_{50} (PC9AR cells), H1975 cells or H1975OR cells: treated with 10k or 10l at 4× IC_{50} (H1975OR cells); (E and F) Migration of NSCLC cells were inhibited by treatment with 10 μM 10k or 10l. Representative images of the wound-healing assay are shown (right panels), along with quantitative analysis of migration rates as a percentage relative to the $t = 0$ time point (left panels). Statistical significance: *P*-values was set as follows: $^{***}P < 0.001$.

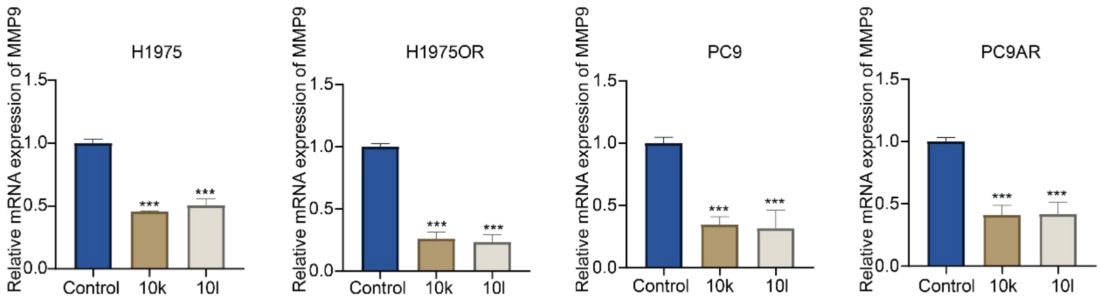

Supplementary Figure 7. The mRNA expression levels of MMP9 were analyzed by RT-qPCR. NSCLC cells were treated with each compound for 24 h. After treatment, total RNA was extracted and analyzed using RT-qPCR. PC9 cells or PC9AR cells: treated with 10k or 10l at 1× IC_{50} (PC9 cells), H1975 cells or H1975OR cells: treated with 10k or 10l at 1× IC_{50} (H1975 cells). Data are expressed as mean ± SD, with statistical significance: *P*-values were set as follows: $^{***}P < 0.001$ ($n = 3$).

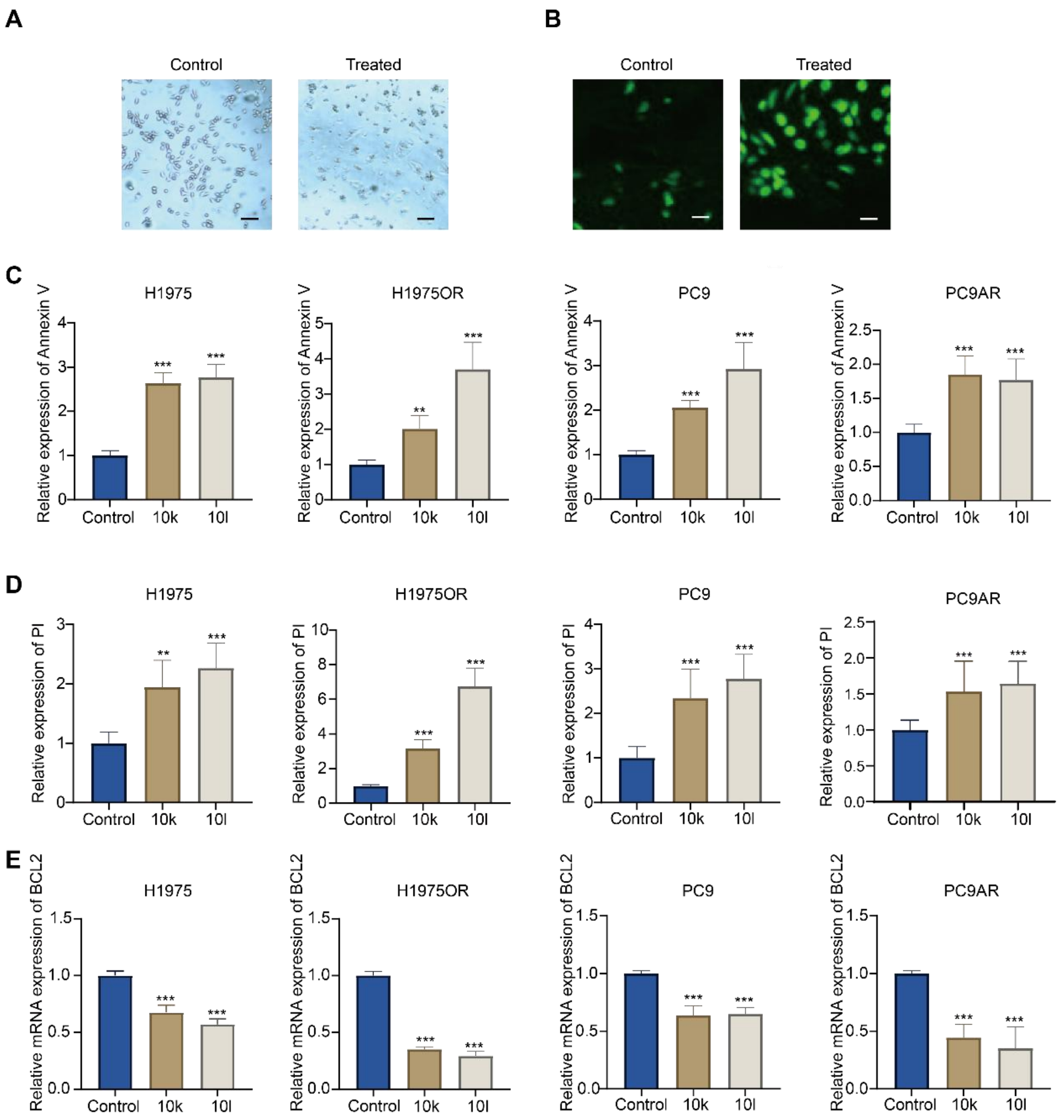

Supplementary Figure 8. The new FAK inhibitors 10k and 10l induce apoptosis and necrosis in EGFR-TKI-sensitive and -resistant NSCLC cells. (A) Representative bright-field images of NSCLC cells treated with 10k. Scale bar: 100 µm; (B) Representative fluorescence microscopy images showing Annexin V levels in NSCLC cells after treatment with 10k. Left panel: untreated control cells; right panel: cells treated with 10k. Scale bar: 20 µm; The levels of Annexin V (C) and PI (D) was assessed using fluorescence analysis. H1975 cell or H1975OR cells: treated with 10k or 10l at 2× IC_{50} (H1975 cells), PC9 cells or PC9AR cells: treated with 10k or 10l at 1.5× IC_{50} (PC9 cells); (E) The expression of BCL2 mRNA was measured using RT-qPCR. PC9 cell or PC9AR cells: treated with 10k or 10l at 1× IC_{50} (PC9 cells), H1975 cell or H1975OR cell: treated with 10k or 10l at 1× IC_{50} (H1975 cells). Statistical significance: *P*-values were set as follows: $^{**}P < 0.01$, $^{***}P < 0.001$.

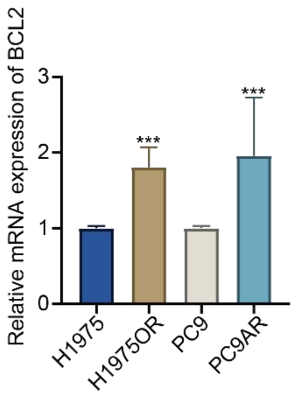

Supplementary Figure 9. mRNA expression levels of BCL2 in H1975, H1975OR, PC9, and PC9AR cells were analyzed using RT-qPCR. Data are presented as mean ± SD, with statistical significance: *P*-values was set as follows: $^{***}P < 0.001$ ($n = 3$).

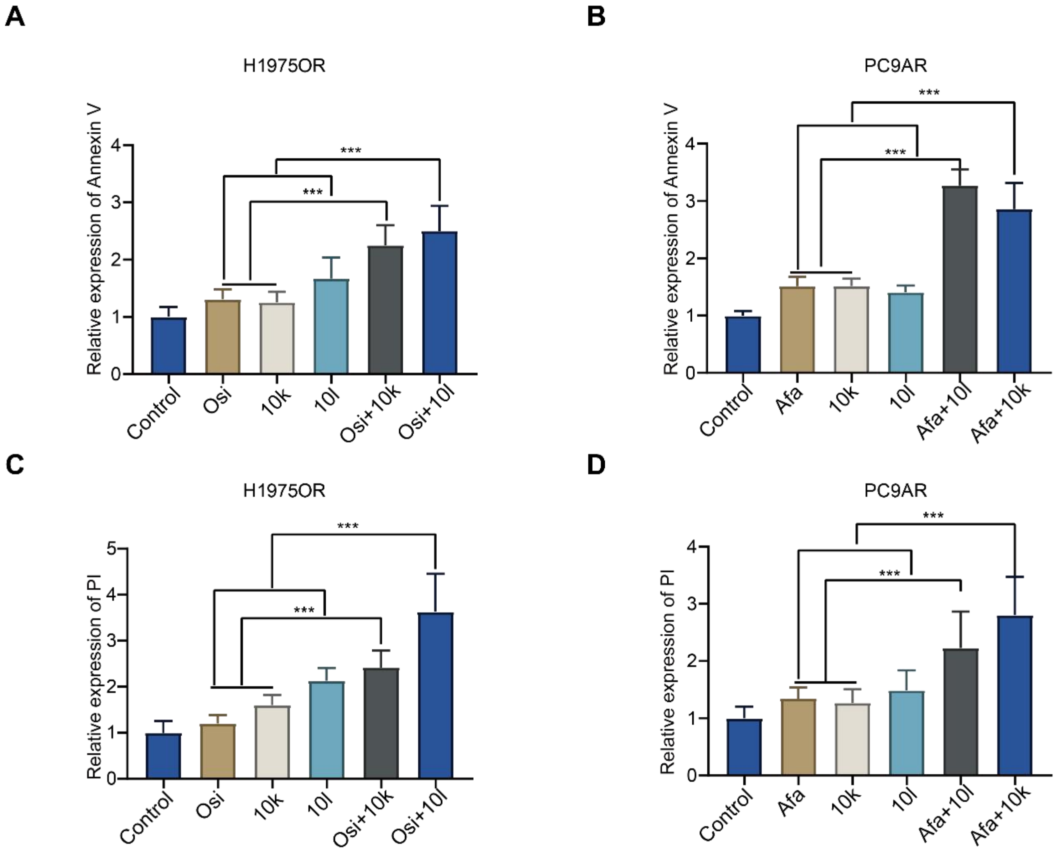

Supplementary Figure 10. The combination of 10k, 10l, and EGFR-TKIs effectively induces cell death. (A-D) PC9AR and H1975OR cells were treated for 2 days with indicated compounds alone, or their combinations. The levels of Annexin V and PI were assessed using fluorescence-based analysis. PC9AR cells: treated with Afatinib at 0.25 × IC_{50} (PC9AR cells) and 10k or 10l at 1× IC_{50} (PC9AR cells) or combination, H1975OR cells: treated with Osimertinib at 1× IC_{50} (H1975OR cells) and 10k,10I at 1× IC_{50} (H1975OR cells) or combination. Afa, Afatinib; Osi, Osimertinib. Statistical significance: *P*-values was set as follows: $^{***}P < 0.001$.

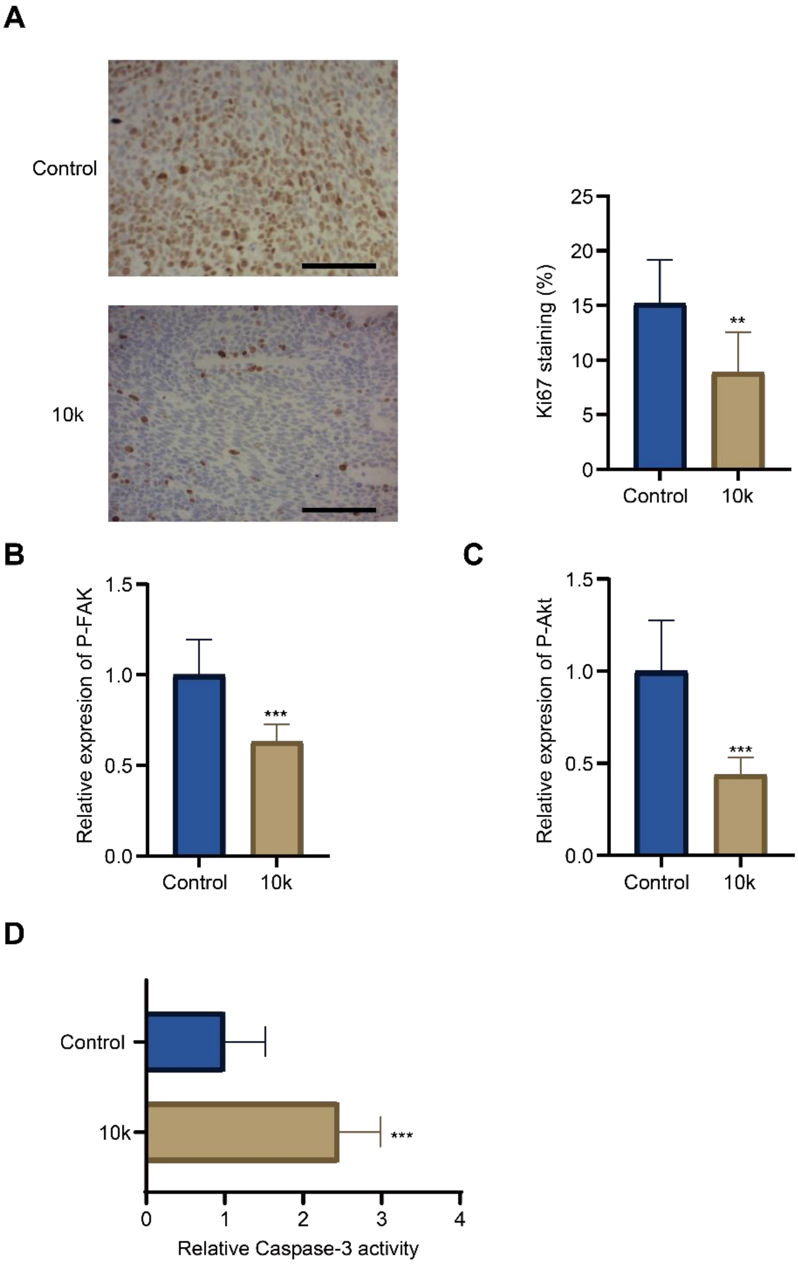

Supplementary Figure 11. The novel FAK inhibitor 10k downregulates Ki-67, p-FAK, and p-Akt expression and promotes apoptosis in subcutaneous xenograft H1975OR tumors. (A) Representative images and quantification of Ki-67 expression (right panel) in tumor sections from resected subcutaneous xenografts in control (upper left panel) and 10k-treated (lower left panel) groups. Scale bar = 100 µm; ELISA-based quantification of (B) p-FAK and (C) p-Akt levels in tumor lysates from resected subcutaneous xenografts treated with 10k compared with controls; (D) Quantification of cleaved caspase-3 activity, measured by ELISA in tumor lysates from the same xenografts, comparing 10k-treated and control groups. Data are presented as mean ± SD. Statistical significance: $^{**}P < 0.01$, $^{***}P < 0.001$ ($n = 3$).

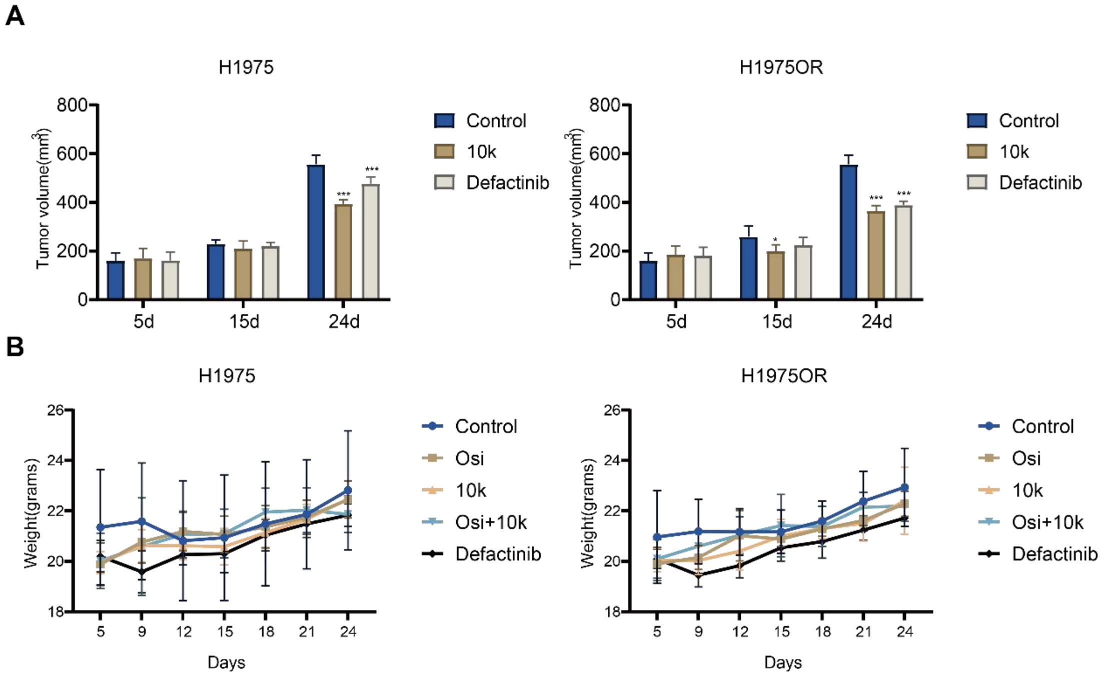

Supplementary Figure 12. Evaluation of the inhibitory effects of 10k and Defactinib in vivo. (A) Tumor growth in mice bearing H1975 (left panel) or H1975OR (right panel) tumors treated with 10k (20 mg/kg) or Defactinib (50 mg/kg) for 2 weeks. Tumor volumes were measured and calculated at specified time points to assess the efficacy of the treatments; (B) Body weight of mice was monitored throughout the treatment period to evaluate potential systemic toxicity. Afa, Afatinib; Osi, Osimertinib. Data are presented as mean ± SD, with statistical significance: *P*-values were set as follows: $^{*}P < 0.05$, $^{***}P < 0.001$ ($n = 6$).

REFERENCES

1. Pecoraro C, Carbone D, Scianò F, et al. Exploring the therapeutic potential of a novel series of imidazothiadiazoles targeting focal adhesion kinase (FAK) for pancreatic cancer treatment: synthesis, mechanistic insights and promising antitumor and safety profile. *J Drug Target.* 2024; 32: 1278-1294. DOI: 10.1080/1061186x.2024.2385557.
2. Kuiper JL, Hashemi SM, Thunnissen E, et al. Non-classic EGFR mutations in a cohort of Dutch EGFR-mutated NSCLC patients and outcomes following EGFR-TKI treatment. *Br J Cancer.* 2016; 115: 1504-1512. DOI: 10.1038/bjc.2016.372.
3. van Veggel B, de Langen AJ, Hashemi S, et al. Crizotinib treatment for patients with EGFR mutation positive NSCLC that acquire cMET amplification after EGFR TKI therapy results in short-lived and heterogeneous responses. *Lung Cancer.* 2018; 124: 130-134. DOI: 10.1016/j.lungcan.2018.07.030.
4. Che P, Gregori A, Bergonzini C, et al. Differential Sensitivity to Ionizing Radiation in Gemcitabine-Resistant and Paclitaxel-Resistant Pancreatic Cancer Cells. *Int J Radiat Oncol Biol Phys.* 2024; 118: 1328-1343. DOI: 10.1016/j.ijrobp.2023.10.035.
5. Pacchiana R, Mullappilly N, Pinto A, et al. 3-Bromo-Isoxazoline Derivatives Inhibit GAPDH Enzyme in PDAC Cells Triggering Autophagy and Apoptotic Cell Death. *Cancers (Basel).* 2022; 14: 3153. DOI: 10.3390/cancers14133153.
6. Massihnia D, Avan A, Funel N, et al. Phospho-Akt overexpression is prognostic and can be used to tailor the synergistic interaction of Akt inhibitors with gemcitabine in pancreatic cancer. *J Hematol Oncol.* 2017; 10: 9. DOI: 10.1186/s13045-016-0371-1.
7. Churchman ML, Evans K, Richmond J, et al. Synergism of FAK and tyrosine kinase inhibition in Ph(+) B-ALL. *JCI Insight.* 2016; 1: e86082. DOI: 10.1172/jci.insight.86082.
8. Detre S, Saclani Jotti G and Dowsett M. A "quickscore" method for immunohistochemical semiquantitation: validation for oestrogen receptor in breast carcinomas. *J Clin Pathol.* 1995; 48: 876-878. DOI: 10.1136/jcp.48.9.876.
9. Vallés-Martí A, Mantini G, Manoukian P, et al. Phosphoproteomics guides effective low-dose drug combinations against pancreatic ductal adenocarcinoma. *Cell Rep.* 2023; 42: 112581. DOI: 10.1016/j.celrep.2023.112581.

10. Giovannetti E, Funel N, Peters GJ, et al. MicroRNA-21 in pancreatic cancer: correlation with clinical outcome and pharmacologic aspects underlying its role in the modulation of gemcitabine activity. *Cancer Res.* 2010; 70: 4528-4538. DOI: 10.1158/0008-5472.Can-09-4467.
11. Bononi G, Di Stefano M, Poli G, et al. Reversible Monoacylglycerol Lipase Inhibitors: Discovery of a New Class of Benzylpiperidine Derivatives. *J Med Chem.* 2022; 65: 7118-7140. DOI: 10.1021/acs.jmedchem.1c01806.
12. Jensen LJ, Kuhn M, Stark M, et al. STRING 8—a global view on proteins and their functional interactions in 630 organisms. *Nucleic Acids Res.* 2008; 37: D412-D416. DOI: 10.1093/nar/gkn760 %J
13. Ogata H, Goto S, Sato K, et al. KEGG: Kyoto Encyclopedia of Genes and Genomes. *Nucleic Acids Res.* 1999; 27: 29-34. DOI: 10.1093/nar/27.1.29.
14. Huang D, Sherman BT and Lempicki RA. Systematic and integrative analysis of large gene lists using DAVID bioinformatics resources. *Nat Protoc.* 2009; 4: 44-57. DOI: 10.1038/nprot.2008.211.
15. Mingoia F, Di Sano C, D'Anna C, et al. Synthesis of new antiproliferative 1,3,4-substituted-pyrrolo[3,2-c]quinoline derivatives, biological and in silico insights. *Eur J Med Chem.* 2023; 258: 115537. DOI: 10.1016/j.ejmech.2023.115537.
16. Madhavi Sastry G, Adzhigirey M, Day T, et al. Protein and ligand preparation: parameters, protocols, and influence on virtual screening enrichments. *J Comput Aided Mol Des.* 2013; 27: 221-234. DOI: 10.1007/s10822-013-9644-8.
17. Banks JL, Beard HS, Cao Y, et al. Integrated Modeling Program, Applied Chemical Theory (IMPACT). *J Comput Chem.* 2005; 26: 1752-1780. DOI: org/10.1002/jcc.20292.
18. Berger BT, Amaral M, Kokh DB, et al. Structure-kinetic relationship reveals the mechanism of selectivity of FAK inhibitors over PYK2. *Cell Chem Biol.* 2021; 28: 686-698.e687. DOI: 10.1016/j.chembiol.2021.01.003.
19. Sherman W, Beard HS and Farid R. Use of an Induced Fit Receptor Structure in Virtual Screening. *Chem Biol Drug Des.* 2006; 67: 83-84. DOI: 10.1111/j.1747-0285.2005.00327.x.
20. Sherman W, Day T, Jacobson MP, et al. Novel Procedure for Modeling Ligand/Receptor Induced Fit Effects. *J Med Chem.* 2006; 49: 534-553. DOI: 10.1021/jm050540c.
21. Percie du Sert N, Ahluwalia A, Alam S, et al. Reporting animal research:

Explanation and elaboration for the ARRIVE guidelines 2.0. *PLoS Biol.* 2020; 18: e3000411. DOI: 10.1371/journal.pbio.3000411.

22. Giovannetti E, Wang Q, Avan A, et al. Role of CYB5A in pancreatic cancer prognosis and autophagy modulation. *J Natl Cancer Inst.* 2014; 106: djt346. DOI: 10.1093/jnci/djt346.

23. Cavazzoni A, La Monica S, Alfieri R, et al. Enhanced efficacy of AKT and FAK kinase combined inhibition in squamous cell lung carcinomas with stable reduction in PTEN. *Oncotarget.* 2017; 8: 53068-53083. DOI: 10.18632/oncotarget.18087.

24. Zavrakidis I, Jóźwiak K and Hauptmann M. Statistical analysis of longitudinal data on tumour growth in mice experiments. *Sci Rep.* 2020; 10: 9143. DOI: 10.1038/s41598-020-65767-7.
